# Risk prediction of QTc prolongation occurrence in cancer patients treated with commonly used oral tyrosine kinase inhibitors: machine learning modeling or conventional statistical analysis better?

**DOI:** 10.1186/s12911-025-03091-8

**Published:** 2025-08-15

**Authors:** Hsiang-Wen Lin, Tien-Chao Lin, Chien-Ning Hsu, Tzu-Pei Yeh, Yu-Chieh Chen, Liang-Chih Liu, Chen-Yuan Lin

**Affiliations:** 1 School of Pharmacy and Graduate Institute, China Medical University, Taichung, Taiwan; 2 Department of Pharmacy, China Medical University Hospital, Taichung, Taiwan; 3 Department of Pharmacy System, Outcomes and Policy, College of Pharmacy, University of Illinois at Chicago, Chicago, Illinois USA; 4 Department of Pharmacy, Kaohsiung Chang Gung Memorial Hospital, Kaohsiung, Taiwan; 5 School of Pharmacy, Kaohsiung Medical University, Kaohsiung, Taiwan; School of Nursing, China Medical University, Taichung, Taiwan; 6 School of Nursing, China Medical University, Taichung, Taiwan; 7 Surgical Department, China Medical University Hospital, Taichung, Taiwan; 8 College of Medicine, China Medical University, Taichung, Taiwan; 9 Division of Hematology and Oncology, China Medical University Hospital, No. 100, Section 1, Jingmao Road, Beitun District, Taichung City, 406040 Taiwan

**Keywords:** QTc prolongation, Oral, Tyrosine kinase inhibitors, Logistic regression, Machine learning, Algorithm

## Abstract

**Background:**

Cancer patients receiving targeted therapies need to prevent QTc prolongation and life-threatening cardiovascular (CV) events to maintain a balanced benefit-risk ratio. This study aimed to develop an optimal prediction model for QTc prolongation risk and estimate its risk probability in cancer patients treated with oral tyrosine kinase inhibitors (TKIs).

**Methods:**

This retrospective cohort study analyzed electronic medical records (EMR) of cancer patients newly treated with commonly used oral TKIs at a medical center between January 2016 and December 2020. QTc prolongation was defined as ≥ 450 ms in males and ≥ 470 ms in females using Bazett’s formula. The study followed four key steps: (1) Managing missing data, (2) Identifying important variables, (3) Training and testing the best prediction models, (4). Estimating risk probability and determining cut-off points. Both univariate logistic regression (LR) and supervised machine learning (ML) approaches were used for variable selection. The backward LR method and seven ML algorithms were applied to train and test the prediction models. The best model was identified based on model performance, fitting criteria, area under the receiver operating characteristic curve (AUROC), risk probability cut-off points, and clinical relevance.

**Results:**

The statistical 12-parameter model demonstrated excellent performance (AUROC = 0.89, sensitivity = 0.91, specificity = 0.75) and strong discrimination ability for risk probability prediction (AUROC = 0.78, cut-off = 0.46), outperforming other ML models. In the final best model: the baseline risk probability of QTc prolongation was 0.13, even in the absence of other contributing factors. Baseline QTc prolongation and a history of cardiovascular disease (excluding arrhythmia, cardiomyopathy, etc.) contributed the most to incremental risk probability (0.471 and 0.282, respectively), after controlling for other factors. The remaining 10 factors each contributed to an increased probability of QTc prolongation for more than 0.14 probability.

**Conclusions:**

A logistic regression model utilizing 12 easily accessible variables from EMRs outperformed ML models in predicting the risk probability of QTc prolongation in cancer patients newly treated with five oral TKIs. These findings serve as a valuable clinical reference for integrating digital monitoring into cardiovascular care for cancer survivors undergoing targeted therapy with TKIs. They also underscore the importance of screening baseline ECG before initiating TKIs to assess the risk of QTc prolongation, facilitating early intervention and prevention in the future.

**Supplementary information:**

The online version contains supplementary material available at 10.1186/s12911-025-03091-8.

## Background

Patients with cancer are at a higher risk of dying from non-cancer diseases; cardiovascular (CV) diseases contribute the most to this risk (about 22%), around 2 times higher than general population [1, 2]. Given CV-related death gradually outweighs cancer-related death as cancer survivors live longer [3, 4], it is necessary to balance the harm of CV-related side effects with the benefits of new therapies for cancer patients, especially those who had pre-existing CV diseases, and/or shared similar risk factors [5]. QTc prolongation is one of primary safety indicators related to arrhythmia risk or sudden cardia-death (SCD) among patients with cancer and who receive targeted therapies, including tyrosine kinase inhibitors (TKIs) [6, 7]. Unlike conventional cytotoxic chemotherapy, which indiscriminately affects both healthy and cancerous cells and primarily prepared as intravenous dosage forms, TKIs represent an important class of targeted therapies focusing on specific molecular alterations essential for cancer cell growth and metastasis. They not only enhance the efficacy of cancer treatment but are also designed as oral formulations, typically resulting in fewer side effects that usually do not overlap with those of traditional cytotoxic chemotherapy (e.g., including cardiovascular adverse events) [6–8]. The incidence of QTc prolongation among cancer patients receiving these targeted therapies has been reported to be as high as 42%. Therefore, corresponding preventive measures and appropriate management strategies in clinical practice warrant particular attention [6, 7].

Approximately one-third of patients receiving TKI therapies developed QTc prolongation (defined as ≥ 450 millisecond (ms) in men and ≥ 470 ms in women based on Fridericia formula) [7], and 5% of the events were considered as life-threatening complications. Vascular endothelial growth factor inhibitors (VEGF*i*_s_) and BCR-ABL inhibitors (BCR-ABL*i*_s_) are two of the most frequent used TKIs that have a risk of QTc prolongation [9]. TKIs have multiple targets and can affect cardiomyocytes by altering complexities signaling (including tyrosine kinase activity) and leading to on– or off-targets that block cardiac channels and lead to TKI-related cardiotoxicities [10] (including QTc prolongation [11], or life-threatening arrhythmia, such as torsade de points [TdP] [12]).

A risk score obtained from multivariate logistic regressions (LR) was developed and validated to predict QT prolongation (defined as QTc > 500 ms or incremental > 60 ms than baseline) for patients in the cardiac critical care units at the university-affiliated tertiary care hospital in the United States in 2013 [13]. Given the incident QT prolongation was about 30% in the cardiac settings, the final risk score composed with 10 variables (e.g., age, gender, diagnoses of acute myocardial infarction, sepsis or heart failure, treated with QTc prolongation medications or loop diuretic, and potassium level). The risk model demonstrated significant predictive value, with an area under the receiver operating characteristic curve (AUROC) of 0.832. It also showed balanced sensitivity and specificity, both approximately 0.7 for the high-risk category (score ≥ 11), where the weight scoring system was determined based on logistic regression coefficients (≤ 0.44 = 1 point, 0.45 to 0.94 = 2 points, ≥ 0.95 = 3 points) [13]. Later, the RISQ-PATH algorithm was further used to categorize the risk of QTc prolongation for a large patient cohort in both out- and in-patient medical settings in Belgium in 2018 [14]. While its optimal prediction model had high AUROC and sensitivity (0.77 and 0.87), its specificity (0.46) was not as good and the model was developed through LR as well [14]. Thus far, none of prediction model of QTc prolongation is available to perform routine assessments in electronic medical records (EMR) for patients with cancer new treatments (targeted therapies or immune-check point inhibitors [ICI]) and/or traditional chemotherapies [15], yet.

A few studies have explored the application of machine learning (ML) (e.g., deep neural networks and random forest [RF]) and LR approaches using EMR data to develop risk prediction model that could be used to identify patients with risk of drug-induced QTc prolongation [16, 17]. The baseline QTc interval, C-reactive protein (CRP) level, heart rate at baseline, age, serum calcium level, renal function, serum potassium level, and the atrial fibrillation status were the important features in the ML models to predict drug-induced QTc prolongation, whereas the selected ML approaches performed better than LR [16]. However, these studies only focused on non-cardiac drugs with the potential to prolong the QT intervals [17] for patients in ambulatory or acute care settings [13–17], but not for the new treatments of cancer therapy in the outpatient settings.

Given that oral TKIs are commonly used for various stable outpatients with cancer, this study aimed to develop the risk prediction model of QTc prolongation through conventional statistical LR or ML algorithms and its risk probability estimation for cancer patients newly treated with the commonly used oral TKIs, in terms of VEGF*i*_s_ and BCR-ABL*i*_s_.

## Methods

### Study design and variables of interest

We designed a retrospective cohort study to retrieve the data from EMR based on the published studies, including the variables used in the RISQ-PATH models [13, 14] and the other factors with pathophysiological or biological relevance of QTc prolongation or of cardiotoxicity among cancer patients [18–30]: factors associated with drug-induced QTc interval prolongation (demographics, pre-existing cardiovascular diseases, neurological diseases, medications prone to lead to QTc prolongation, smoking, alcohol abuse, body mass index [BMI], hypertension, liver and/or renal dysfunction, endocrine diseases) [14, 19–30], other related co-morbidities listed in Table S1 (including hypo- or hyper-thyroidism) and cancer statues (cancer types, cancer performance status scale [Eastern Cooperative Oncology Group, ECOG]), usage of the individual TKI on the index date (including defined daily dose [DDD], which assumes an average maintenance dose per day for a drug used for its main indication in adults provided by World Health Organization) [31], relevant co-medications (Table S2, e.g., listed as CredibeMeds [18], and drugs prone to encounter drug-drug interaction [e.g., due to strong cytochrome P450 3A4 (CYP3A4) inhibitors might extend the exposure of some TKIs]), electrocardiography (ECG) findings, and laboratory data (e.g., serum electrolytes, albumin, CRP, hemoglobin [Hb], lactate dehydrogenase [LDH], aspartate aminotransferase [AST], alanine aminotransferase [ALT]). Particularly, those CV diseases, excluding the stand-alone CV diseases which were strongly associated with QTc prolongation (including arrythmia [ICD 10 as I44 - I49], ischemia heart disease, hypertension, heart failure, cardiomyopathy), were grouped together (named as “other CV diseases”). All these variables were considered as potential independent variables or features in the following modeling analysis.

### Clinical settings and patient characteristics

This study was conducted in a tertiary medical center in central Taiwan with a relatively high-volume in offering cardiac and cancer care (CMUH). We focused on all consecutive patients with cancer who were newly treated with the most commonly used oral TKIs, including VEGF*i*s (sunitinib and sorafenib) and BCR-ABL*i*s (imatinib, nilotinib,and dasatinib), regardless of their cancer types, from January 2016 to December 2020. Importantly, there were none of other oral TKI (MAPK/ERK Kinase [MEK] inhibitors, BRAF inhibitors, Janus Kinase [JAK] inhibitors) available in CMUH at that time. We included patients with baseline QTc interval within 1 year prior to the index date but excluded those patients with a QTc interval < 240 ms or > 760 ms, younger than 18 years of age, with an artificial pacemaker, or without ECG findings within 6 months after the index date.

The index date was the initial date that patients treated with one of the aforementioned commonly oral TKIs. We assessed the ECG findings of each patient within a 6-month observation period after the index date (Figure S1). The end date was either the date with the longest QTc interval within the 6-month period, the last date of the 6-month period, or last date of the oral TKI prescription up to December 31, 2020. We calculated the QTc interval based on Bazett’s formula [32]. Specifically, the dependent variable, QTc prolongation, was defined as ECG showed ≥ 450 ms for male patients and ≥ 470 ms for female patients [25].

### Data collection and processing

We performed the descriptive analysis for demographic or cancer data observed on the index date, the ECG findings at different time points, co-morbidities or medical history within 1 year prior to the index date, co-medications within 7-days prior to the index date, and lab data (most recent values within 3 months) (Figure S1 and Table S3). We randomly divided all eligible patients into training and testing datasets (70% and 30%) and compared the differences between datasets by using the *chi*-square and *t* tests. We employed single imputation using the average (for BMI only) or regression multivariate imputation approach for each dataset (replaced with the maximum expected value for other variables) in the two datasets separately. We compared continuous variables with independent *t* tests to ensure comparability before and after imputation. We performed univariate analyses using LR analysis in the training dataset to identify the statistical important variables (with *p*_s_ < 0.05). The odds ratios and 95% confidence intervals (CIs) were sorted before and after imputation of the missing data. In addition, we performed sensitivity analyses to examine the robustness of the statistically significant variables used to train the prediction model.

### Statistical analysis

#### Model training

**Fig. 1:**
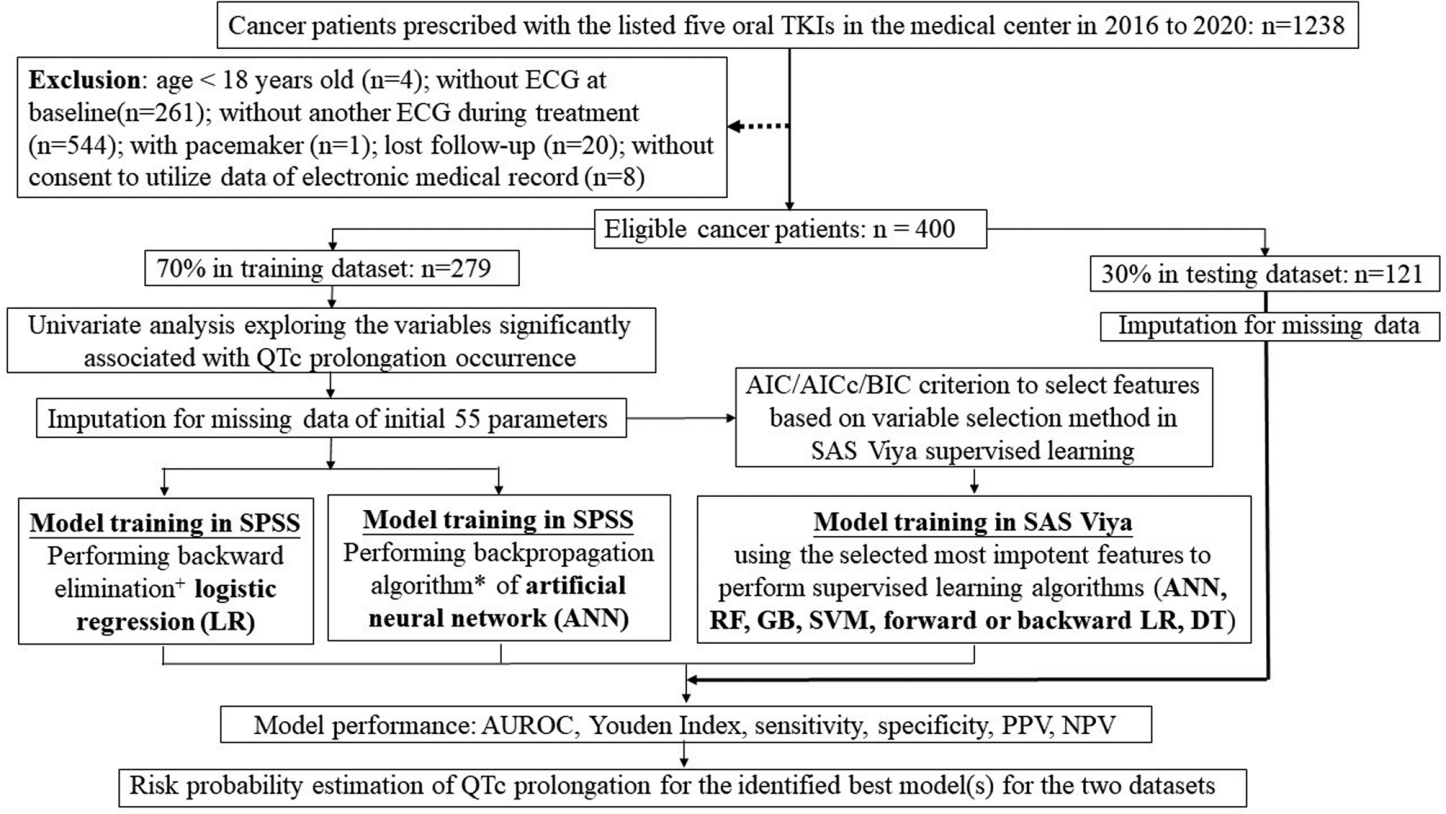
Study flow of data analysis. TKI = tyrosine kinase inhibitors; QTc = Corrected QT Interval; ECG = electrocardiography; AIC = Akaike information criterion; AICc = Akaike information criterion corrected for small sample (AICc); BIC = Bayesian Information Criterion; MSE = mean squared error; AUROC = Area Under Receiver Operating Characteristics Curve; PPV = positive predictive value; NPV = negative prediction value (NPV); ML = machine learning; ANN = artificial neural network; RF = random forests; GB = gradient boosting; SVM = support vector machine; LR = logistic regression; DT = decision trees. ^**+**^ The predictor with the highest *p* value was eliminated first, repeated the process to exclude one variable at one time until the last variable without setting up stop criterion as the default *p* value ≥ 0.1 in SPSS. ^*^The order of variable elimination performed in standard backpropagation algorithm of artificial neural network was the same as that performed of backward elimination logistic regression

First, we performed the conventional statistical analyses (i.e., backward LR) for all initial 55 variables and developed models in the training dataset. The variable with the highest *p* value was eliminated first, and the process was repeated to exclude one variable at one time until the last variable remained (Fig. 1). Whenever the AUROC reaches the maximum (more than 0.5 [as random guess] up to 1.0 (perfect accuracy), there were greater probabilities of obtaining prediction models with good performance. Then, the standard backpropagation algorithm of an artificial neural network (ANN) was performed, where the elimination order of variables was the same as that process in the prior backward LR. All these analyses were performed in SPSS Statistics Version 25.

Next, we performed ML approach to select a set of important variables by performing forward-swap and stepwise LR based on Akaike information criterion (AIC), the Akaike information criterion corrected for a small sample (AICc), and the Bayesian information criterion (BIC) in SAS Viya supervised learning (as SAS machine learning program). This process yielded the mean squared error (MSE) between the prediction and observation model after selecting the corresponding variables into the model. Then, we trained and identified the best model by performing seven supervised learning algorithms (ANN, RF, gradient boosting (GB), support vector machine (SVM), forward and backward LR, and decision trees (DT)) for the final set of most important variables with the smallest AIC, AICc or BIC in SAS Viya, accordingly.

#### Model comparisons

We assessed the model performances through conducting Receiver Operating Characteristics Curve (ROC curve) analysis and compared its AUROC obtained from the training dataset, as well as the accuracy, Youden index (i.e., sensitivity + specificity-1), sensitivity, specificity, positive predictive value (PPV) and negative prediction value (NPV) obtained from the testing dataset. Ideally, the higher AUROC, better sensitivity and specificity was considered as the better models. Accordingly, the corresponding best model derived from statistical and ML approach were compared for their clinical implication of QTc prolongation. We further explored the model fitting criteria (AIC, BIC, − 2 log likelihood, where least is a better model) and feasibility of the risk probability derived from the best prediction models by performing standardized multivariate LR analysis.

#### Risk probability estimation of QTc prolongation

Then, we calculated the predicted risk probability for the best *i*-parameter model(s) with available coefficients derived from the standardized multivariate LR findings, as the follows:

Predicted risk probability (*P*) = exp^z^ /(1 + exp^z^), where z is intercept + β_1_ × X_1_ **+** β_2_ × X_2_ **+** … … + β_i_ × X_i_ and *i* is the number of parameters identified in the final best models.

With the risk probability ranged from 0 to 1, we utilized the occurrence of QTc prolongation as the state variable to determine the cut-off point, which could be used to differentiate the high- or low-risk probability of QTc prolongation in the best models by performing ROC curve analysis. We compared the AUROC and its 95% CI, sensitivity, and specificity for the identified cut-off points in both datasets. In addition, the predicted risk probability of QTc prolongation for the individual variable, whenever controlling for the other variables (replace X = 1 into the equation for an individual coefficient and X = 0 for the other variables), in the best models were compared and ranked to appreciate the impact extent and clinical meaning of the individual variables. Further, we performed subgroup analyses considering the impact of certain variables to examine the robustness of the risk probability estimation.

## Results

### Characteristics of study cohort

Of all 1,238 consecutive patients enrolled in the original cohort, 400 were eligible for further analysis. The majority of them were male, diagnosed with liver cancer (specifically hepatocellular carcinoma [HCC]) and had stage III or IV disease (Table S3). Sorafenib, a VEGF*i*, was used more frequently than the other four oral TKIs. The average electrolyte levels were within the normal ranges but the liver functions and cancer-related biomarkers were higher than the normal ranges. We found 23% and 41.5 % of patients showed QTc prolongation at the baseline and by the end date, respectively. The patterns of characteristics and variables of interest in the training and testing datasets (n = 279 and 121, respectively) were comparable (all *p*_s_ > 0.05 in Table S4). The continuous variables were not significantly different before and after imputation (Table S5), although the missing patterns were different among the variables.

### Model development and validation

In the conventional statistical analyses, the 16 most significant variables (*p*_s_ < 0.05) were identified almost the same before and after imputation (Table S6 and S7). The AUROC was 0.89 and 0.90 for the backward LR and ANN models, respectively, after deleting 43 non-significant variables (Table 1). The 12-parameters model (named as statistical best model) derived from the LR algorithm composed the 12 most significant variables in EMR and easily present the associations with the outcome than the model derived from the ANN algorithm. While more than 81% of final eligible patients were diagnosed with HCC, the 9 statistically significant variables were similar in the prediction models for patients with and without HCC (Table S8).

**Table 1:** The model performance either developed by backward elimination logistic regression (LR) or standard backpropagation algorithm of artificial neural network (ANN) in testing dataset

		Logistic Regression (LR)						Artificial Neural Network (ANN)					
**Deleted a variable at one time**	**No. of para.**	**AU** **ROC**	**Sen.**	**Spe.**	**Youden**	**PPV**	**NPV**	**AU** **ROC**	**Sen.**	**Spe.**	**Youden**	**PPV**	**NPV**
**None (using all 55 variables)**	**55**	**0.82**	**0.77**	**0.67**	**0.44**	**0.74**	**0.71**	**0.88**	**0.88**	**0.67**	**0.55**	**0.76**	**0.82**
Acute renal failure	54	0.83	0.76	0.67	0.43	0.74	0.70	0.90	0.88	0.67	0.55	0.76	0.82
Concomitant compound Rx cause abnormal potassium	53	0.82	0.77	0.67	0.44	0.74	0.71	0.87	0.76	0.81	0.57	0.83	0.74
Hepatitis C	52	0.83	0.76	0.67	0.43	0.74	0.70	0.88	0.77	0.78	0.55	0.81	0.74
ALT ≥ 120 IU/L	51	0.83	0.76	0.65	0.41	0.72	0.69	0.89	0.94	0.60	0.54	0.74	0.89
Magnesium < 1.7 mmol/L	50	0.82	0.77	0.65	0.42	0.73	0.71	0.87	0.89	0.71	0.60	0.79	0.85
Ever used other TKI before	49	0.82	0.77	0.65	0.42	0.73	0.71	0.89	0.85	0.75	0.60	0.80	0.80
Cardiomyopathy	48	0.84	0.79	0.69	0.48	0.75	0.73	0.89	0.89	0.67	0.56	0.77	0.84
Diastolic blood pressure ≥ 90 mmHg	47	0.84	0.79	0.69	0.48	0.75	0.73	0.89	0.94	0.76	0.70	0.83	0.91
Heart surgery history	46	0.84	0.80	0.69	0.49	0.76	0.75	0.87	0.91	0.67	0.58	0.77	0.86
Use VEGFR currently	45	0.84	0.80	0.69	0.49	0.76	0.75	0.89	0.91	0.71	0.62	0.79	0.87
Hepatitis B	44	0.84	0.79	0.71	0.50	0.76	0.74	0.89	0.88	0.73	0.61	0.79	0.83
Diabetes mellitus	43	0.84	0.79	0.71	0.50	0.76	0.74	0.87	0.91	0.69	0.60	0.78	0.86
Systolic blood pressure ≥ 140 mmHg	42	0.85	0.80	0.71	0.51	0.77	0.75	0.90	0.86	0.73	0.59	0.79	0.82
Endocrine diseases	41	0.85	0.79	0.71	0.50	0.76	0.74	0.89	0.77	0.82	0.59	0.84	0.75
Liver damage (upon ICD)	40	0.85	0.80	0.71	0.51	0.77	0.75	0.89	0.94	0.67	0.61	0.78	0.90
eGFR < 30 mL/min	39	0.86	0.80	0.71	0.51	0.77	0.75	0.88	0.83	0.76	0.59	0.81	0.79
Liver transplantation	38	0.86	0.80	0.71	0.51	0.77	0.75	0.88	0.88	0.80	0.68	0.84	0.85
Heart failure	37	0.86	0.80	0.71	0.51	0.77	0.75	0.90	0.82	0.84	0.66	0.86	0.79
Days of using the TKI ≥ 60 days	36	0.86	0.80	0.71	0.51	0.77	0.75	0.88	0.89	0.67	0.56	0.77	0.84
With HTN diagnosis	35	0.86	0.79	0.71	0.50	0.76	0.74	0.90	0.89	0.65	0.54	0.76	0.84
Alcohol habit	34	0.86	0.79	0.71	0.50	0.76	0.74	0.89	0.88	0.76	0.64	0.82	0.84
Hematology cancer	33	0.86	0.80	0.71	0.51	0.77	0.75	0.88	0.89	0.67	0.56	0.77	0.84
Diagnosed with hypertension on end day	32	0.87	0.80	0.71	0.51	0.77	0.77	0.91	0.86	0.71	0.57	0.78	0.81
Moderate- severe chronic kidney disease	31	0.87	0.83	0.71	0.54	0.77	0.78	0.90	0.86	0.84	0.70	0.86	0.84
DDD of TKI on end date ≥ 1	30	0.87	0.83	0.73	0.56	0.79	0.78	0.89	0.85	0.78	0.63	0.82	0.81
Use possible risk CredibleMeds ≥ 1 item	29	0.87	0.82	0.75	0.57	0.79	0.77	0.90	0.88	0.75	0.63	0.81	0.84
With metastasis	28	0.87	0.83	0.75	0.58	0.80	0.79	0.88	0.85	0.78	0.63	0.82	0.81
Smoking habit	27	0.87	0.82	0.75	0.57	0.79	0.77	0.91	0.89	0.75	0.64	0.81	0.85
Hb < 9 g/dL	26	0.88	0.85	0.71	0.56	0.78	0.80	0.88	0.83	0.73	0.56	0.79	0.78
LDH ≥ 225 U/L	25	0.87	0.85	0.71	0.56	0.78	0.80	0.90	0.88	0.80	0.68	0.84	0.85
AFP ≥ 9 ng/mL	24	0.86	0.79	0.73	0.52	0.78	0.74	0.88	0.86	0.75	0.61	0.80	0.82
DDD of the TKI on index date ≥ 1	23	0.87	0.80	0.73	0.53	0.78	0.75	0.88	0.86	0.76	0.62	0.81	0.82
CRP > 10 mg/L	22	0.87	0.82	0.75	0.57	0.79	0.77	0.89	0.91	0.69	0.6	0.78	0.86
Concomitant use Rx cause hyperkalemia	21	0.88	0.82	0.75	0.57	0.79	0.77	0.90	0.86	0.76	0.62	0.81	0.82
Recurrent of cancer	20	0.88	0.83	0.75	0.58	0.80	0.79	0.89	0.91	0.73	0.64	0.80	0.87
Age ≥ 45 when initiated TKI	19	0.89	0.86	0.73	0.59	0.79	0.82	0.89	0.89	0.73	0.62	0.80	0.85
Ischemia heart disease	18	0.88	0.86	0.75	0.61	0.80	0.80	0.88	0.88	0.88	0.76	0.82	0.84
Neurological disease(s)	17	0.88	0.86	0.75	0.61	0.80	0.82	0.90	0.86	0.78	0.64	0.83	0.83
BMI ≥ 27	16	0.88	0.89	0.75	0.64	0.81	0.85	0.89	0.89	0.80	0.69	0.84	0.86
Sodium < 135 mmol/L	15	0.89	0.89	0.76	0.65	0.82	0.86	0.90	0.89	0.76	0.65	0.82	0.86
Use high risk Rx of CredibleMeds ≥ 1 item	14	0.88	0.89	0.75	0.64	0.81	0.85	0.90	0.86	0.76	0.62	0.81	0.82
Concomitant use Rx cause hypokalemia	13	0.88	0.89	0.73	0.62	0.80	0.85	0.89	0.88	0.76	0.64	0.82	0.84
**Male gender**	**12**	**0.89**	**0.91**	**0.75**	**0.66**	**0.81**	**0.87**	**0.90**	**0.91**	**0.78**	**0.69**	**0.83**	**0.88**
Arrythmia	11	0.89	0.91	0.75	0.66	0.81	0.87	0.90	0.85	0.76	0.61	0.81	0.81
Concurrent use medications ≥ 5 items	10	0.90	0.89	0.71	0.60	0.79	0.85	0.90	0.89	0.76	0.65	0.82	0.86
Other CV diseases	9	0.89	0.89	0.67	0.56	0.77	0.84	0.90	0.86	0.80	0.66	0.84	0.83
INR > 1.5	8	0.89	0.89	0.67	0.56	0.77	0.84	0.89	0.91	0.75	0.66	0.81	0.87
ECOG score ≥ 3	7	0.88	0.89	0.71	0.60	0.79	0.85	0.89	0.85	0.80	0.65	0.84	0.81
AST ≥ 105 IU/L	6	0.87	0.91	0.69	0.60	0.78	0.86	0.87	0.85	0.73	0.58	0.79	0.80
Check ECG in emergency room	5	0.86	0.89	0.67	0.56	0.77	0.84	0.88	0.85	0.85	0.70	0.80	0.80
Albumin < 3.8 g/dL	4	0.86	0.91	0.69	0.60	0.78	0.86	0.86	0.91	0.71	0.62	0.79	0.87
Total bilirubin > 2 mg/dL	3	0.81	0.91	0.65	0.56	0.76	0.86	0.81	0.91	0.65	0.56	0.76	0.86
Calcium < 8 mmol/L	2	0.78	0.91	0.65	0.56	0.76	0.86	0.78	0.91	0.65	0.56	0.76	0.86
Potassium < 3.5 mmol/L	1	0.72	0.92	0.51	0.43	0.69	0.85	0.72	0.92	0.51	0.43	0.69	0.85
Prolongation of QTc in the baseline	0	-	-	-	-	-	-	-	-	-		-	-

OR = odds ratio; QTc = Corrected QT Interval; AST = aspartate aminotransferase; ECOG = Eastern Cooperative Oncology Group Performance Status Scale; INR = International Normalized Ratio; Rx = prescribed medication; BMI = body mass index; TKI = tyrosine kinase inhibitor; CRP = C-reactive protein; DDD = defined daily dose (The assumed average maintenance dose per day for a drug used for its main indication in adults https://www.who.int/tools/atc-ddd-toolkit/about-ddd); AFP = alpha-fetoprotein; LDH = lactate dehydrogenase; Hb = hemoglobin; HTN = hypertension; eGFR = estimated Glomerular filtration rate; ICD = The International Statistical Classification of Diseases and Related Health Problems 10th Revision; VEGF = Vascular Endothelial Growth Factor inhibitor; ALT = alanine aminotransferase; AUROC = Area Under Receiver Operating Characteristics Curve; Sen. = sensitivity; Spe. = specificity; PPV = positive predictive value; NPV = negative prediction value (NPV)

#Other CV diseases = those CV diseases, excluding the stand-alone CV diseases which were strongly related to QTc prolongation (i.e., arrythmia [ICD 10 as I44 - I49], ischemia heart disease, hypertension, heart failure, cardiomyopathy), were grouped together to compare.

*the order of variable elimination performed in standard backpropagation algorithm of artificial neural network (ANN) is the same as that performed of backward elimination logistic regression (LR) (i.e., deleted a variable at one time begin with the least significant variable).

In the ML approach, stepwise and forward LR identified the exact same set of variables in the training dataset (Table S9). We selected the most “important” 12 variables (with the lowest AIC) as the inputs for supervised learning of seven algorithms in the training dataset. Finally, LR algorithm, both forward or backward LR, produced the best accuracy (0.84) and Youden index (0.68) (named as ML best model) (Table 2). The 12-feature ANN algorithm performed similarly to that of the LR algorithm. However, the optimization numerical methods and other hyperparameter settings of the ANN were different for the two sets of 12 variables in the two models performed either in SPSS or SAS Viya, respectively (Table S10).

**Table 2:** The model performance developed by 7 supervised machine learning algorithms in the testing dataset for the identified 12 most important features

ML Model	Accuracy	AUROC	Youden index	Sen.	Spe.	PPV	NPV
Forward LR	0.84	0.86	0.68	0.80	0.88	0.85	0.84
Backward LR	0.84	0.86	0.68	0.80	0.88	0.85	0.84
ANN	0.83	0.89	0.67	0.84	0.83	0.81	0.86
SVM	0.83	0.87	0.64	0.73	0.91	0.87	0.80
RF	0.81	0.89	0.62	0.93	0.70	0.72	0.92
GB	0.80	0.85	0.55	0.85	0.70	0.70	0.85
DT	0.74	0.72	0.43	0.51	0.92	0.85	0.69

LR = logistic regression LR; ANN = artificial neural network, SVM = support vector machine, RF = random forest, GB = gradient boosting, DT = decision tree; ROC curve = Receiver Operating Characteristics Curve; AUROC = Area Under Receiver Operating Characteristics Curve; Sen. = sensitivity; Spe. = specificity; PPV = positive predictive value; NPV = negative prediction value (NPV). Youden index = Sensitivity + Specificity – 1

### Model performances

**Fig. 2:**
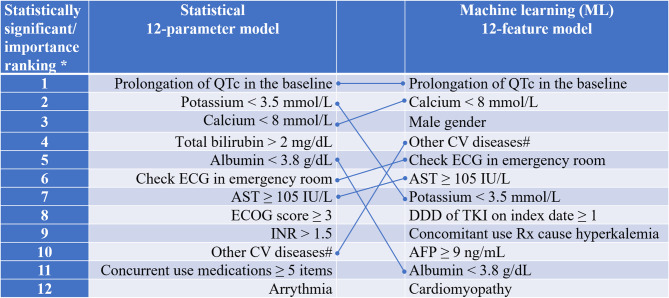
Ranking of factors in the best model either obtained from statistical model or machine learning model. QTc = Corrected QT Interval; AST = aspartate aminotransferase; ECOG = Eastern Cooperative Oncology Group Performance Status Scale; INR = International Normalized Ratio; Rx = prescribed medication; BMI = body mass index; TKI = tyrosine kinase inhibitor; CRP = C-reactive protein; AFP = alpha-fetoprotein; DDD = defined daily dose (The assumed average maintenance dose per day for a drug used for its main indication in adults https://www.who.int/tools/atc-ddd-toolkit/about-ddd); TKI = tyrosine kinase inhibitor. *1 ~ 12 = most to least significant/ important factor. #Other CV diseases = those CV diseases, excluding the stand-alone CV diseases which were strongly related to QTc prolongation (i.e., arrythmia [ICD 10 as I44 - I49], ischemia heart disease, hypertension, heart failure, cardiomyopathy), were grouped together to compare. –: Seven factors were in common in the two different sets of 12 factors for the corresponding best models either developed by the statistically driven model or machine learning model

After comparing the two sets of 12 variables in the best model derived from either statistical or ML approach, respectively, there were seven variables in common (Fig. 2). The other four variables selected through ML approach (i.e., DDD of TKI on index date ≥ 1, concomitant use medications associated with hyperkalemia, AFP ≥ 9 ng/ml, cardiomyopathy) in the training dataset were relatively less important and also not listed as the statistical associated factors (Table S7). The statistical best model not only performed better with least AIC, BIC and − 2 Log Likelihood by performing standardized multivariate LR, but also was more clinical meaningful than the ML best model (Table 3). Further, the 7-parameter model with the common variables in statistical and ML models was developed and tested with even better model fitting and more parsimonious (Table S11).

**Table 3:** Performance of risk probability estimation of QTc Prolongation in the best model either obtained from statistical or machine learning approach in the training dataset

	Statistical 12-parameter model		ML 12-feature model	
	**Model Fitting Criteria of Standardized Logistic Regression**			
	AIC	188.278		211.603
	BIC	235.484		258.809
	−2 Log Likelihood	162.278		185.603
**Variable (X**_**i**_**)**	**Variable definition**	**Coefficient (β**_**i**_**)**	**Variable definition**	**Coefficient (β**_**i**_**)**
	**Intercept**	**−2.039**	**Intercept**	**−2.115**
1	Prolongation of QTc in the baseline	1.922	Prolongation of QTc in the baseline	2.294
2	Potassium < 3.5 mmol/L	0.859	Calcium < 8 mmol/L	1.253
3	Calcium < 8 mmol/L	0.275	Male gender	1.269
4	Total bilirubin > 2 mg/dL	0.959	Other CV diseases	1.818
5	Albumin < 3.8 g/dL	0.365	Check ECG in emergency room	0.490
6	Check ECG in emergency room	0.479	AST ≥ 105 IU/L	0.882
7	AST ≥ 3 105 IU/L	0.592	Potassium < 3.5 mmol/L	0.983
8	ECOG score ≥ 3	0.821	DDD of TKI on index date ≥ 1	−0.721
9	INR > 1.5	0.821	Concomitant use Rx cause hyperkalemia	0.774
10	Other CV diseases^#^	1.103	AFP ≥ 9 ng/mL	−0.606
11	Concurrent use medications ≥ 5 items	0.408	Albumin < 3.8 g/dL	0.519
12	Arrythmia	0.589	Cardiomyopathy	−2.556
Cut-off point of predicted risk probability*		**0.463**^**+**^^1^		**0.414**^+3^
	AUROC (95 % CI)	0.784 (0.729–0.840)	AUROC (95 % CI)	0.834 (0.785–0.882)
	Sensitivity	0.667	Sensitivity	0.721
	Specificity	0.815	Specificity	0.815

ML = machine learning; QTc = Corrected QT Interval; AST = aspartate aminotransferase; ECOG = Eastern Cooperative Oncology Group Performance Status Scale; INR = International Normalized Ratio; Rx = prescribed medication; BMI = body mass index; TKI = tyrosine kinase inhibitor; CRP = C-reactive protein; AFP = alpha-fetoprotein; DDD = defined daily dose (The assumed average maintenance dose per day for a drug used for its main indication in adults https://www.who.int/tools/atc-ddd-toolkit/about-ddd); TKI = tyrosine kinase inhibitor

#Other CV diseases = those CV diseases, excluding the stand-alone CV diseases which were strongly related to QTc prolongation (i.e., arrythmia [ICD 10 as I44 - I49], ischemia heart disease, hypertension, heart failure, cardiomyopathy), were grouped together to compare. *predicted risk probability of QTc prolongation within 6 months among cancer patients newly treated with selected 5 oral TKIs = exp ^z^ /(1 + exp ^z^), where z = intercept + β_1_ × X_1_**+** β_2_ × X_2_**+** β_3_ × X_3_**+** β_4_ × X_4_**+** β_5_ × X_5_**+** β_6_ × X_6_**+** … … + β_i_ × X_*i*_, (*i* = number of parameters being identified in the final best model), where exp refers exponential function. ^+1^106 patients in training dataset (38%) had a predicted risk probability of QTc prolongation as 0.463 or higher based upon the statistical 12-parameter model; 112 patients in training dataset (40.1%) had a predicted risk probability of QTc prolongation occurrence as 0.414 or higher based upon the ML 12-feature model

### Predicted risk probability of QTc prolongation

The discrimination ability of the predicted risk probability for the two best models, as well as the 7-parameter model, in the training dataset were comparable (AUROC = 0.784 versus 0.791 in Table 3 and 0.791 in Table S11). However, statistical best model was more rigorous to differentiate high- or low-risk of QTc prolongation (cut-off risk probability ≥ 0.463 with 38% categorized as high-risk) than ML best model (0.414 and 40.1%, respectively). Further, the statistical best model performed better to differentiate the risk levels in the testing dataset than in training dataset (AUC: 0.89 vs. 0.78) but their cut-off points were similar (around 0.46 in Table S12). Further subgroup analysis findings (e.g., TKI_Sorafenib *versus* TKI_not sorafenib or HCC cancer *versus* Not-HCC cancer) also inferred the robustness of statistical best model to estimate the risk probability for those patients with different characteristics in both datasets (all AUROC > 0.7). Further, the cut-off predicted risk probability derived from the parsimonious 7-parameter model was relatively lower in the testing dataset but not observed in the ML best model.

Given there were more than 0.115 chance to occur QTc prolongation without exposing to the other associated factors for the statistical best model (0.108 for ML best model), those patients with experience of QTc prolongation in baseline or diagnosed with the other CV diseases might occur relative more incremental risk probability after controlling for the other factors in these models, including in the 7-parameter model (Table 4 and Table S11). These two factors even contributed more than the cut-off risk probability in the ML best model (0.545 and 0.426 ≥ 0.414, respectively). Patients with QTc prolongation in the baseline was relative more risk (0.471 ≥ 0.463) based on the findings in the statistical best model. Then, the extent and ranking of the predicted risk probability for the remaining 10 factors were various in the two best models. In particular, the other 10 factors also contributed more than 0.14 chance in the statistical best model but not for those least important factors in the ML best model (AFP ≥ 9 ng/mL, DDD of TKI on index date ≥ 1, Cardiomyopathy were all < 0.1).

**Table 4:** Predated risk probability of QTc prolongation whenever controlling for the other factors in the best model obtained from statistical or machine learning approach in the training dataset

Statistical 12-parameter model		ML 12-feature model	
**Variable and its definition**	**risk probability**^**1**^** (ranking)**	**Variable definition**	**risk probability**^**1**^** (ranking)**
Intercept	0.115	Intercept	0.108
Prolongation of QTc in the baseline	0.471 (1)	Prolongation of QTc in the baseline	0.545 (1)
Other cardiovascular diseases^#^	0.282 (2)	Other cardiovascular diseases	0.426 (2)
Total bilirubin > 2 mg/dL	0.254 (3)	Male gender	0.300 (3)
Potassium < 3.5 mmol/L	0.235 (4)	Calcium < 8 mmol/L	0.297 (4)
ECOG score ≥ 3	0.228 (5)	Potassium < 3.5 mmol/L	0.244 (5)
INR > 1.5	0.228 (5)	AST ≥ 105 IU/L	0.226 (6)
Arrythmia	0.190 (7)	Concomitant use Rx cause hyperkalemia	0.207 (7)
AST ≥ 105 IU/L	0.190 (7)	Albumin < 3.8 g/dL	0.169 (8)
Check ECG in emergency room	0.174 (9)	Check ECG in emergency room	0.165 (9)
Concurrent use medications ≥ 5 items	0.164 (10)	AFP ≥ 9 ng/mL	0.062 (10)
Albumin < 3.8 g/dL	0.158 (11)	DDD of TKI on index date ≥ 1	0.055 (11)
Calcium < 8 mmol/L	0.146 (12)	Cardiomyopathy	0.009 (12)

ML = machine learning; QTc = Corrected QT Interval; CV = cardiovascular disease; AST = aspartate aminotransferase; ECOG = Eastern Cooperative Oncology Group Performance Status Scale; INR = International Normalized Ratio; Rx = prescribed medication; BMI = body mass index; TKI = tyrosine kinase inhibitor; CRP = C-reactive protein; AFP = alpha-fetoprotein; DDD = defined daily dose (The assumed average maintenance dose per day for a drug used for its main indication in adults https://www.who.int/tools/atc-ddd-toolkit/about-ddd); TKI = tyrosine kinase inhibit

^1^ predicted risk probability of QTc prolongation within 6 months among cancer patients newly treated with oral TKIs = exp ^z^ /(1 + exp ^z^), where z = intercept + β_1_*X_1_**+** β_2_*X_2_**+** β_3_*X_3_**+** β_4_*X_4_**+** β_5_*X_5_**+** β_6_*X_6_**+** … … + β_i_*X_*i*_, (*i* = number of parameters being identified in the final best model), where exp refers exponential function. The risk probability was derived after transfer the individual coefficient (X = 1) in the equation and controlling for the other factors (X = 0)

Ranking: 1 = highest risk probability when controlling for the other factors, 7 or 12 = least risk probability

#Other CV diseases = those CV diseases, excluding the stand-alone CV diseases which were strongly related to QTc prolongation (i.e., arrythmia [ICD 10 as I44 - I49], ischemia heart disease, hypertension, heart failure, cardiomyopathy), were grouped together to compare

Thus, the statistical 12-parameter model is considered as the final best model to estimate risk probability of QTc prolongation. Accordingly, the predicted probability (*P*) of experiencing QTc prolongation (QTc ≥ 450 ms for males or ≥ 470 ms for females) among cancer patients initially treated with five common oral TKIs (X) can be calculated using the following formula:

$$P = \frac{{\exp \left( z \right)}}{{1 + \exp \left( z \right)}}$$

Where

z = − 2.039 + 1.922 × *X*_1_ **+** 0.859 × *X*_2_ **+** 0.275 × *X*_3_ + 0.959 × *X*_4_ + 0.365 × *X*_5_ + 0.479 × *X*_6_ + 0.592 × *X*_7_ + 0.821 × *X*_8_ + 0.821 × *X*_9_ + 1.103 × *X*_10_ + 0.408 × *X*_11_ + 0.589 × *X*_12_

Each variable (*X*_i_) is assigned a value of 1 if the condition is met, or 0 otherwise: *X*_1_ = QTc prolongation at baseline; *X*_2_ = Potassium < 3.5 mmol/L; *X*_3_ = Calcium < 8 mmol/L; *X*_4_ = Albumin < 3.8 g/dL; *X*_5_ = AST ≥ 105 IU/L; *X*_6_ = ECG performed in the emergency room; *X*_7_ = Presence of cardiovascular diseases excluding those strongly associated with QTc prolongation (e.g., arrhythmia, ischemic heart disease, hypertension, heart failure, cardiomyopathy); *X*_8_ = Total bilirubin > 2 mg/dL; *X*_9_ = ECOG score ≥ 3; *X*_10_ = Concurrent use of ≥ 5 medications; *X*_11_ = INR > 1.5; *X*_12_ = Presence of arrhythmia

Finally, an Excel-based calculator (provided as a supplementary excel file) has been developed to facilitate efficient computation by importing patient data. This tool can be integrated into clinical decision support systems in hospitals or used in community healthcare apps or website to facilitate real-time risk assessment.

## Discussion

This is the first study to explore the best prediction model of QTc prolongation based on routine EMR data by applying conventional statistical LR with ML modeling for cancer patients newly treated with the commonly used oral VEGF*i*_s_ and BCR-ABL*i*_s_. The statistical best model with 12 easily-accessible variables developed by the LR algorithm showed better model performance, discrimination of risk probability, and clinical meaning to predict QTc prolongation risk than the other ML models.

The factors identified in the best model closely aligned with those listed in the review of common clinical risk factors for drug-induced QT prolongation and TdP in patients receiving anti-cancer drugs. These included cardiovascular diseases, liver disease, medication use, and electrolyte imbalances [33]. However, unlike the reviewed risk factors, this study also incorporated baseline QTc prolongation, cancer-related liver biomarkers, and cancer performance status, which were not previously considered. Only four identified variables (QTc prolongation at baseline, potassium, calcium, arrythmia) are part of the risk factors listed in the “smart algorithm” based on the optimized RISQ-PATH model for patients treated with non-cardiac drugs whenever they visited hospitals in Belgium (Table S13) [14]. In particular, the identified poor hepatic factors were consistent with findings from other studies that examined associations between QT interval prolongation and liver biomarkers [34, 35]. Patel’s study reported similar risk factors, such as higher INR and lower serum albumin levels for patients with end-stage liver diseases [34]. Additionally, data from the National Health and Nutrition Examination Survey (NHANES) highlighted an association between increasing total bilirubin levels and QT prolongation [35]. Unfortunately, none of these studies enrolled cancer patients.

Further, the corresponding cut-off risk probability in this study was pretty higher than those patients involved in the study of optimized RISQ-PATH model (i.e., exposed to the listed non-cardiac medications associated with high-risk QTc prolongation (≥0.463 *versus* 0.035) [14]. Such findings mached with the prior studies showing that 30% to 42% of patients receiving TKIs or cancer target therapies might occur QTc prolongation [6, 7]. Specifically, our study confirmed that those patients with prior experience of QTc prolongation and existing the other CV diseases (e.g., myocarditis, bradycardia [other than well-known arrythmia or so]), who were similar as “who had pre-existing CV diseases, and/or shared similar risk factors” [5], should be monitored even more intensively to prevent QTc prolongation for cancer survivors newly treated with oral TKIs. The other cancer related variables (e.g., ECOG score, total bilirubin, AST, INR) also need to be paid more attention other than those well-known factors (e.g., potassium, arrythmia) for those cancer patients treated with targeted therapies in clinical practice in the future.

While ML approaches using data with complex images and in EMR are becoming more prevalent [36], we found that the statistical LR algorithm provided the best prediction in the final best model. We believe that the statistical approach to identify significant variables in SPSS is better than the automatic variable selection approach in SAS Viya supervised learning. In fact, not all the variable selection methods can be performed in the commonly used statistical software (i.e., SAS, SPSS, and R). SPSS offers relatively fewer variable selection methods than the others [37]. However, Heinze et al recommended that the conventional variable selection method (i.e., LR) might be unstable and produce heterogenous results regarding the selection of important variables as compared with the modern variable selection methods (e.g., the least absolute shrinkage and selection operator [LASSO] approach) [37] as that in SAS Viya supervised learning.

Two studies demonstrated that the developed prediction models through ML approaches performed better than that through LR approach for patients susceptible to drug-induced QT prolongation using EMR data [16, 17]. However, none of the selected features were disclosed and interpreted in the risk prediction model [16], and the authors considered only one oral TKI, Vandetanib, and the definition of QTc prolongation was more rigorous (>500 ms) [17]. We focused on outpatients with cancer who were newly treated with the commonly used oral TKIs but Simon et al [17] focused on those inpatients used the 33 listed medications in CredibleMedS.org [18]. In addition, the ML best model in our study considered comprehensive parameters with a relatively small sample and performed better than these two aforementioned ML-related studies (accuracy = 0.84 and AUROC = 0.86 *vs* accuracy = 0.82 or AUCROC = 0.71, respectively) [16, 17].

Our study addresses a literature gap regarding the prediction of QTc prolongation using EMR data for cancer patients using common oral TKIs and those who have a relative high tendency of QTc prolongation and cardiotoxicity [11–14]. Given that digital health is implemented more frequently in modern society, our study provides evidence to consider applying this final best model in the clinical decision support system (CDSS) in EMR or being integrated into patients’ wearable devices that allow physicians, pharmacists and/or nurses to identify or screening patients with a high risk of QTc interval prolongation in advance accordingly. We believe these approaches could provide signals to guide further intensive monitoring to prevent TdP and SCD from happening in the future.

Our study has some limitations that must be considered. First, several study features must be noted: its retrospective nature, potential information biases for the EMR data, and it was performed at a single medical center in central Taiwan. Our datasets also have inherent imperfections in data collection (e.g., missing information), so imputation was required to perform ML analysis. Second, the sample size was not as large as the previous study [17], but our final best model performed better than that study. Further, the generalizability of our results is still limited because the data were consecutively obtained from those cancer patients treated with the five oral TKIs at CMUH during 5-year period only. Further, our population seemed predominantly diagnosed with HCC given the prevalence of hepatitis B or C was relatively high in Taiwan. In contrast, more than 46% of eligible patients treated with TKIs—primarily imatinib (a BCR-ABL*i*) and pazopanib (a VEGF*i*)—at the Mayo Clinic in the U.S. were mainly diagnosed with renal cell carcinoma (RCC) [7]. Notably, none of the enrolled patients were diagnosed with liver cancer. Indeed, the associated factors of QT prolongation among patients with HCC were different from that those without HCC. However, our further sensitivity analysis to explore the prediction performance of the best models showed similar patterns for the data either from patients with or without HCC.

Third, the selected ML approaches of variable selection methods may not be sufficiently robust. The penalized regression framework for modern variable selection methods (e.g., LASSO) may be a better choice to improve stability and prediction [37]. However, the stepwise selection regression model among the supervised variable selection methods in ML was considered as the best method to select the fewest and most important features whenever predicting the overall equipment effectiveness in Yilmaz’s study [38]. To reduce the effects of different procedures in the common used programs (SPSS and SAS Viya) for variable selection, we chose the conventional statistical variable selection method rather than LASSO in SAS Viya to identify a set of variables that jointly explain the maximum variances as that performed in SPSS. We believe the variable selection approaches might contribute to the differences between the two sets of 12 identified variables in the different modeling programs.

Fourth, two key factors—baseline QTc prolongation and emergency ECG checks—were identified as significant predictors in both the statistical 12-parameter model and the machine learning 12-feature model. Notably, baseline QTc prolongation emerged as the strongest predictor, with an incremental risk probability of 0.471, exceeding the high-risk cutoff of 0.463. In contrast, urgent ECG checks had a less significant impact in the final best model. This finding aligns with the RISQ-PATH algorithm [14], further reinforcing the predictive role of prior QTc prolongation. Among the 23% of patients with QTc prolongation at baseline, our data do not allow us to definitively determine the underlying cause in all cases. This represents a limitation, as unmeasured factors—such as genetic predisposition or subclinical cardiac conditions—may contribute to the observed prevalence. However, other factors, including electrolyte imbalances (potassium, calcium), liver function markers (bilirubin, INR, AST, albumin), and cardiac comorbidities (e.g., arrhythmia), also played a crucial role in enhancing the predictive accuracy of our statistical 12-parameter model. Overall, this model outperformed others, particularly in terms of AUROC, sensitivity, specificity, and model-fitting criteria, including AIC and − 2 log likelihood (Table 1 and Table 3). However, these findings are associative rather than causal and should be interpreted with caution, as they are based on statistical and data-driven analyses. In clinical practice, QTc prolongation in TKI-treated patients is often nonspecific and incidentally detected during cardiac assessments. Nevertheless, these results underscore the importance of baseline ECG screening before initiating TKIs and highlight the need for regular monitoring to assess QTc prolongation risk, enabling early intervention and prevention.

Finally, the definitions, eligibility, and chosen variables might contribute to various parameter selection and model training. Nevertheless, further comparisons and validation of using the final best model prospectively to explore its application in EMR or wearable devices, as well as further to include more oral targeted therapies (i.e., other available TKs, including MEK inhibitors, BRAF inhibitors, or ICI) or implement this optimal model in various practice settings are needed.

## Conclusions

The statistical 12- parameter model developed by the logistic regression algorithm was considered as the final optimal model to predict the occurrence of QTc prolongation for those cancer patients treated with new and the commonly used five oral TKIs. This model composed of 12 easily-accessible variables from electronic medical records and performed better than the other machine learning models after considering its performance and clinical meaning. The estimated risk probability for QTc prolongation, using a threshold of ≥ 0.46 to define as high risk, revealed good discriminatory capability. This metric can serve as a valuable reference in clinical practice for identifying cancer survivors who may require cardiovascular care. This might be particularly important for patients newly undergoing common oral targeted therapies with TKIs, helping to better balance the benefit-risk ratio of treatment. Further validation and additional studies are needed to ensure the feasibility and application of incorporating the final optimal model or this risk probability estimation of QTc prolongation into EMRs or wearable devices. This integration aims to improve the detection of signals indicating high-risk patients and enable timely and appropriate managements.

## Electronic supplementary material

Below is the link to the electronic supplementary material.

Supplementary Material 1

Supplementary Material 2

## Data Availability

The deidentified datasets used and/or analyzed during the current study are available from the first author (H.-W. Lin) under the reasonable request.

## Abbreviations

CV: Cardiovascular
TKI: Tyrosine kinase inhibitors
EMR: Electronic medical record
ML: Machine learning
LR: Logistic regression
AUROC: Area under receiver operating characteristics curves
SCD: Sudden cardia-death
VEGFis: Vascular endothelial growth factor inhibitors; torsade de point (TdP)
RF: Random forest
CRP: C-creative protein
IRB: Institutional Review Board
BMI: Body mass index
ECG: Electrocardiography
Hb: Hemoglobin
LDH: Lactate dehydrogenase
AST: Aspartate aminotransferase
ALT: Alanine aminotransferase
CIs: Confidence intervals
ANN: Artificial neural network
AIC: Akaike information criterion
AICc: Akaike information criterion corrected for a small sample
BIC: Bayesian information criterion
MSE: Mean squared error
GB: Gradient boosting
SVM: Support vector machine
DT: Decision trees
ROC: Receiver Operating characteristics
PPV: Positive predictive value
NPV: Negative prediction value
HCC: Hepatocellular carcinoma
LASSO: Least absolute shrinkage and selection operator [LASSO]
CDSS: Clinical decision support system
