# Supplementary material for: Risk prediction of QTc prolongation occurrence in cancer patients treated with commonly used oral tyrosine kinase inhibitors: machine learning modeling or conventional statistical analysis better?
Source: BMC Med Inform Decis Mak. 2025 Aug 15;25:310. doi: 10.1186/s12911-025-03091-8 (PMC12355842; doi:10.1186/s12911-025-03091-8)
Supplement: Supplementary file 2 — Supplementary Material 2 [file 12911_2025_3091_MOESM2_ESM.docx]

**Supplementary information**

.

Table S1. List of concerned co-morbidities……………………………………………………..………………...……. 2

Table S2. List of concerned co-medications…………………………………………………….……………….………10

Figure S1. Study design of retrospective cohort study…………………………………………………………..………11

Table S3. Patients’ characteristics, disease, medication and laboratory data…………………….………………..……..12

Table S4. Comparisons of parameters between that in training and testing dataset……………….………………..…...14

.

Table S5. Comparisons of continuous variables before and after imputation……………………………………..…….16

Table S6. Sorting the variables of interest based on the odds ratio for the data before imputation.………………….…17

Table S7. Sorting the variables of interest based on the odds ratio and *p* value for the data (the most to lest statistically significant) after imputation……………………………………………………………………………………….....….19

Table S8. Univariate logistic regression analysis among patients with hepatocellular carcinoma or not………………………………………………………………………………………………………………………...21

Table S9. 12 variables identified as the most important features through several iterations of feature selection using forward-swap and stepwise regression supervised learning in SAS Viya ……………………………………..……...…23

Table S10. Optimization numerical methods and hyperparameter settings of artificial neural network in SPSS or SAS Viya………………………………………………………………………………………………………………………24

Table S11. Model fitting criteria, risk coefficient, cut-off point of predicted risk probability of QTc prolongation in training dataset for the 7 parameters commonly identified in both best model model…………………………………..25

Table S12. Prediction performance of the best selected models from different model selection and training approaches in training and testing datasets and in different subgroups analysis…………………………………………..….……...26

Table S13. Comparisons of the identified factors in the prediction models obtained from this study and in RISQ-PATH model………………………………………………………………………………………….………………………….27

**Table S1. List of concerned co-morbidities**

| Co-morbidities | ICD-10 |
| --- | --- |
| Myocarditis | I01.2 Acute rheumatic myocarditis  I09.0 Rheumatic myocarditis  I40 Acute myocarditis  I40.0 Infective myocarditis  I40.1 Isolated myocarditis  I40.8 Other acute myocarditis  I40.9 Acute myocarditis, unspecified  I41 Myocarditis in diseases classified elsewhere  I51.4 Myocarditis, unspecified |
| Hypertension | I10 Essential (primary) hypertension  I11 Hypertensive heart disease  I11.0 Hypertensive heart disease with heart failure  I11.9 Hypertensive heart disease without heart failure  I12 Hypertensive chronic kidney disease  I12.0 Hypertensive chronic kidney disease with stage 5 chronic kidney disease or end stage renal disease  I12.9 Hypertensive chronic kidney disease with stage 1 through stage 4 chronic kidney disease, or unspecified chronic kidney disease  I13 Hypertensive heart and chronic kidney disease  I13.0 Hypertensive heart and chronic kidney disease with heart failure and stage 1 through stage 4 chronic kidney disease, or unspecified chronic kidney disease  I13.1 Hypertensive heart and chronic kidney disease without heart failure  I13.10 Hypertensive heart and chronic kidney disease without heart failure, with stage 1 through stage 4 chronic kidney disease, or unspecified chronic kidney disease  I13.11 Hypertensive heart and chronic kidney disease without heart failure, with stage 5 chronic kidney disease, or end stage renal disease  I13.2 Hypertensive heart and chronic kidney disease with heart failure and with stage 5 chronic kidney disease, or end stage renal disease  I15 Secondary hypertension  I15.0 Renovascular hypertension  I15.1 Hypertension secondary to other renal disorders  I15.2 Hypertension secondary to endocrine disorders  I15.8 Other secondary hypertension  I15.9 Secondary hypertension, unspecified |
| Ischemic heart disease | I20 Angina pectoris  I20.0 Unstable angina  I20.1 Angina pectoris with documented spasm  I20.8 Other forms of angina pectoris  I20.9 Angina pectoris, unspecified  I21 Acute myocardial infarction  I21.0 ST elevation (STEMI) myocardial infarction of anterior wall  I21.01 ST elevation (STEMI) myocardial infarction involving left main coronary artery  I21.02 ST elevation (STEMI) myocardial infarction involving left anterior descending coronary artery  I21.09 ST elevation (STEMI) myocardial infarction involving other coronary artery of anterior wall  I21.1 ST elevation (STEMI) myocardial infarction of inferior wall  I21.11 ST elevation (STEMI) myocardial infarction involving right coronary artery  I21.19 ST elevation (STEMI) myocardial infarction involving other coronary artery of inferior wall  I21.2 ST elevation (STEMI) myocardial infarction of other sites  I21.21 ST elevation (STEMI) myocardial infarction involving left circumflex coronary artery  I21.29 ST elevation (STEMI) myocardial infarction involving other sites  I21.3 ST elevation (STEMI) myocardial infarction of unspecified site  I21.4 Non-ST elevation (NSTEMI) myocardial infarction  I22 Subsequent ST elevation (STEMI) and non-ST elevation (NSTEMI) myocardial infarction  I22.0 Subsequent ST elevation (STEMI) myocardial infarction of anterior wall  I22.1 Subsequent ST elevation (STEMI) myocardial infarction of inferior wall  I22.2 Subsequent non-ST elevation (NSTEMI) myocardial infarction  I22.8 Subsequent ST elevation (STEMI) myocardial infarction of other sites  I22.9 Subsequent ST elevation (STEMI) myocardial infarction of unspecified site  I23 Certain current complications following ST elevation (STEMI) and non-ST elevation (NSTEMI) myocardial infarction (within the 28 day period)  I23.0 Hemopericardium as current complication following acute myocardial infarction  I23.1 Atrial septal defect as current complication following acute myocardial infarction  I23.2 Ventricular septal defect as current complication following acute myocardial infarction  I23.3 Rupture of cardiac wall without hemopericardium as current complication following acute myocardial infarction  I23.4 Rupture of chordae tendineae as current complication following acute myocardial infarction  I23.5 Rupture of papillary muscle as current complication following acute myocardial infarction  I23.6 Thrombosis of atrium, auricular appendage, and ventricle as current complications following acute myocardial infarction  I23.7 Postinfarction angina  I23.8 Other current complications following acute myocardial infarction  I24 Other acute ischemic heart diseases  I24.0 Acute coronary thrombosis not resulting in myocardial infarction  I24.1 Dressler's syndrome  I24.8 Other forms of acute ischemic heart disease  I24.9 Acute ischemic heart disease, unspecified  I25 Chronic ischemic heart disease  I25.1 Atherosclerotic heart disease of native coronary artery  I25.10 Atherosclerotic heart disease of native coronary artery without angina pectoris  I25.11 Atherosclerotic heart disease of native coronary artery with angina pectoris  I25.110 Atherosclerotic heart disease of native coronary artery with unstable angina pectoris  I25.111 Atherosclerotic heart disease of native coronary artery with documented spasm  I25.118 Atherosclerotic heart disease of native coronary artery with other forms of angina pectoris  I25.119 Atherosclerotic heart disease of native coronary artery with unspecified angina pectoris  I25.2 Old myocardial infarction  I25.3 Aneurysm of heart  I25.4 Coronary artery aneurysm and dissection  I25.41 Coronary artery aneurysm  I25.42 Coronary artery dissection  I25.5 Ischemic cardiomyopathy  I25.6 Silent myocardial ischemia  I25.7 Atherosclerosis of coronary artery bypass graft(s) and coronary artery of transplanted heart with angina pectoris  I25.70 Atherosclerosis of coronary artery bypass graft(s), unspecified, with angina pectoris  I25.700 Atherosclerosis of coronary artery bypass graft(s), unspecified, with unstable angina pectoris  I25.701 Atherosclerosis of coronary artery bypass graft(s), with documented spasm  I25.708 Atherosclerosis of coronary artery bypass graft(s), unspecified, with other forms of angina pectoris  I25.709 Atherosclerosis of coronary artery bypass graft(s), unspecified, with unspecified angina pectoris I25.71 Atherosclerosis of autologous vein coronary artery bypass graft(s) with angina pectoris  I25.71 Atherosclerosis of autologous vein coronary artery bypass graft(s) with angina pectoris  I25.710 Atherosclerosis of autologous vein coronary artery bypass graft(s) with unstable angina pectoris  I25.711 Atherosclerosis of autologous vein coronary artery bypass graft(s) with documented spasm  I25.718 Atherosclerosis of autologous vein coronary artery bypass graft(s) with other forms of angina pectoris  I25.719 Atherosclerosis of autologous vein coronary artery bypass graft(s) with unspecified angina pectoris  I25.72 Atherosclerosis of autologous artery coronary artery bypass graft(s) with angina pectoris  I25.720 Atherosclerosis of autologous artery coronary artery bypass graft(s) with unstable angina pectoris  I25.721 Atherosclerosis of autologous artery coronary artery bypass graft(s) with documented spasm  I25.728 Atherosclerosis of autologous artery coronary artery bypass graft(s) with other forms of angina pectoris  I25.729 Atherosclerosis of autologous artery coronary artery bypass graft(s) with unspecified angina pectoris  I25.73 Atherosclerosis of nonautologous biological coronary artery bypass graft(s) with angina pectoris  I25.730 Atherosclerosis of nonautologous biological coronary artery bypass graft(s) with unstable angina pectoris  I25.731 Atherosclerosis of nonautologous biological coronary artery bypass graft(s) with documented spasm  I25.738 Atherosclerosis of nonautologous biological coronary artery bypass graft(s) with other forms of angina pectoris  I25.739 Atherosclerosis of nonautologous biological coronary artery bypass graft(s) with unspecified angina pectoris  I25.75 Atherosclerosis of native coronary artery of transplanted heart with angina pectoris  I25.750 Atherosclerosis of native coronary artery of transplanted heart with unstable angina  I25.751 Atherosclerosis of native coronary artery of transplanted heart with documented spasm  I25.758 Atherosclerosis of native coronary artery of transplanted heart with other forms of angina pectoris  I25.759 Atherosclerosis of native coronary artery of transplanted heart with unspecified angina pectoris  I25.76 Atherosclerosis of bypass graft of coronary artery of transplanted heart with angina pectoris  I25.760 Atherosclerosis of bypass graft of coronary artery of transplanted heart with unstable angina  I25.761 Atherosclerosis of bypass graft of coronary artery of transplanted heart with documented spasm  I25.768 Atherosclerosis of bypass graft of coronary artery of transplanted heart with other forms of angina pectoris  I25.769 Atherosclerosis of bypass graft of coronary artery of transplanted heart with unspecified angina pectoris  I25.79 Atherosclerosis of other coronary artery bypass graft(s) with angina pectoris  I25.790 Atherosclerosis of other coronary artery bypass graft(s) with unstable angina pectoris  I25.791 Atherosclerosis of other coronary artery bypass graft(s) with documented spasm  I25.798 Atherosclerosis of other coronary artery bypass graft(s) with other forms of angina pectoris  I25.799 Atherosclerosis of other coronary artery bypass graft(s) with unspecified angina pectoris  I25.8 Other forms of chronic ischemic heart disease  I25.81 Atherosclerosis of other coronary vessels without angina pectoris  I25.810 Atherosclerosis of coronary artery bypass graft(s) without angina pectoris  I25.811 Atherosclerosis of native coronary artery of transplanted heart without angina pectoris  I25.812 Atherosclerosis of bypass graft of coronary artery of transplanted heart without angina pectoris  I25.89 Other forms of chronic ischemic heart disease  I25.9 Chronic ischemic heart disease, unspecified |
| Nonrheumatic mitral valve disorders (Mitral valve prolapse) | I34 Nonrheumatic mitral valve disorders  I34.0 Nonrheumatic mitral (valve) insufficiency  I34.1 Nonrheumatic mitral (valve) prolapse  I34.2 Nonrheumatic mitral (valve) stenosis  I34.8 Other nonrheumatic mitral valve disorders  I34.9 Nonrheumatic mitral valve disorder, unspecified |
| Cardiomyopathy) | I42.0 Dilated cardiomyopathy  I42.1 Obstructive hypertrophic cardiomyopathy  I42.2 Other hypertrophic cardiomyopathy  I43 Cardiomyopathy in diseases classified elsewhere |
| atrioventricular block | I44 Atrioventricular and left bundle-branch block  I44.0 Atrioventricular block, first degree  I44.1 Atrioventricular block, second degree  I44.2 Atrioventricular block, complete  I44.3 Other and unspecified atrioventricular block  I44.30 Unspecified atrioventricular block  I44.39 Other atrioventricular block  I44.4 Left anterior fascicular block  I44.5 Left posterior fascicular block  I44.6 Other and unspecified fascicular block  I44.60 Unspecified fascicular block  I44.69 Other fascicular block  I44.7 Left bundle-branch block, unspecified |
| sinoatrial block | I45 Other conduction disorders  I45.0 Right fascicular block  I45.1 Other and unspecified right bundle-branch block  I45.10 Unspecified right bundle-branch block  I45.19 Other right bundle-branch block  I45.2 Bifascicular block  I45.3 Trifascicular block  I45.4 Nonspecific intraventricular block  I45.5 Other specified heart block  I45.6 Pre-excitation syndrome  I45.8 Other specified conduction disorders  I45.81 Long QT syndrome  I45.89 Other specified conduction disorders  I45.9 Conduction disorder, unspecified |
| cardiac arrest | I46 Cardiac arrest  I46.2 Cardiac arrest due to underlying cardiac condition  I46.8 Cardiac arrest due to other underlying condition  I46.9 Cardiac arrest, cause unspecified |
| atrial fibrillation | I48 Atrial fibrillation and flutter  I48.0 Paroxysmal atrial fibrillation  I48.1 Persistent atrial fibrillation  I48.2 Chronic atrial fibrillation  I48.3 Typical atrial flutter  I48.4 Atypical atrial flutter  I48.9 Unspecified atrial fibrillation and atrial flutter  I48.91 Unspecified atrial fibrillation  I48.92 Unspecified atrial flutter |
| arrhythmias | I49 Other cardiac arrhythmias  I49.0 Ventricular fibrillation and flutter  I49.01 Ventricular fibrillation  I49.02 Ventricular flutter  I49.1 Atrial premature depolarization  I49.2 Junctional premature depolarization  I49.3 Ventricular premature depolarization  I49.4 Other and unspecified premature depolarization  I49.40 Unspecified premature depolarization  I49.49 Other premature depolarization  I49.5 Sick sinus syndrome  Particularly, those CV diseases, excluding the stand-alone CV diseases strongly related to QTc prolongation (i.e., arrythmia [ICD 10 as I44 - I49], ischemia heart disease, hypertension, heart failure, cardiomyopathy), were grouped together to compare. I49.8 Other specified cardiac arrhythmias  I49.9 Cardiac arrhythmia, unspecified |
| congestive heart failure | I50 Heart failure  I50.1 Left ventricular failure, unspecified  I50.2 Systolic (congestive) heart failure  I50.20 Unspecified systolic (congestive) heart failure  I50.21 Acute systolic (congestive) heart failure  I50.22 Chronic systolic (congestive) heart failure  I50.23 Acute on chronic systolic (congestive) heart failure  I50.3 Diastolic (congestive) heart failure  I50.30 Unspecified diastolic (congestive) heart failure  I50.31 Acute diastolic (congestive) heart failure  I50.32 Chronic diastolic (congestive) heart failure  I50.33 Acute on chronic diastolic (congestive) heart failure  I50.4 Combined systolic (congestive) and diastolic (congestive) heart failure  I50.40 Unspecified combined systolic (congestive) and diastolic (congestive) heart failure  I50.41 Acute combined systolic (congestive) and diastolic (congestive) heart failure  I50.42 Chronic combined systolic (congestive) and diastolic (congestive) heart failure  I50.43 Acute on chronic combined systolic (congestive) and diastolic (congestive) heart failure  I50.9 Heart failure, unspecified |
| Kawasaki syndrome | M30.3 Mucocutaneous lymph node syndrome [Kawasaki] |
| bradycardia | R00.1 Bradycardia, unspecified |
| Liver injuries | B15.0 Hepatitis A with hepatic coma  B16.0 Acute hepatitis B with delta-agent with hepatic coma  B16.2 Acute hepatitis B without delta-agent with hepatic coma  B17.11 Acute hepatitis C with hepatic coma  B19.0 Unspecified viral hepatitis with hepatic coma  B19.11 Unspecified viral hepatitis B with hepatic coma  B19.21 Unspecified viral hepatitis C with hepatic coma  I85 Esophageal varices  I85.0 Esophageal varices  I85.00 Esophageal varices without bleeding  I85.01 Esophageal varices with bleeding  I85.1 Secondary esophageal varices  I85.10 Secondary esophageal varices without bleeding  I85.11 Secondary esophageal varices with bleeding  K70 Alcoholic liver disease  K70.0 Alcoholic fatty liver  K70.1 Alcoholic hepatitis  K70.10 Alcoholic hepatitis without ascites  K70.11 Alcoholic hepatitis with ascites  K70.2 Alcoholic fibrosis and sclerosis of liver  K70.3 Alcoholic cirrhosis of liver  K70.30 Alcoholic cirrhosis of liver without ascites  K70.31 Alcoholic cirrhosis of liver with ascites  K70.4 Alcoholic hepatic failure  K70.40 Alcoholic hepatic failure without coma  K70.41 Alcoholic hepatic failure with coma  K70.9 Alcoholic liver disease, unspecified  K71 Toxic liver disease  K71.0 Toxic liver disease with cholestasis  K71.1 Toxic liver disease with hepatic necrosis  K71.10 Toxic liver disease with hepatic necrosis without coma  K71.11 Toxic liver disease with hepatic necrosis with coma  K71.2 Toxic liver disease with acute hepatitis  K71.3 Toxic liver disease with chronic persistent hepatitis  K71.4 Toxic liver disease with chronic lobular hepatitis  K71.5 Toxic liver disease with chronic active hepatitis  K71.50 Toxic liver disease with chronic active hepatitis without ascites  K71.51 Toxic liver disease with chronic active hepatitis with ascites  K71.6 Toxic liver disease with hepatitis, not elsewhere classified  K71.7 Toxic liver disease with fibrosis and cirrhosis of liver  K71.8 Toxic liver disease with other disorders of liver  K71.9 Toxic liver disease, unspecified  K72 Hepatic failure, not elsewhere classified  K72.0 Acute and subacute hepatic failure  K72.00 Acute and subacute hepatic failure without coma  K72.01 Acute and subacute hepatic failure with coma  K72.1 Chronic hepatic failure  K72.10 Chronic hepatic failure without coma  K72.11 Chronic hepatic failure with coma  K72.9 Hepatic failure, unspecified  K72.90 Hepatic failure, unspecified without coma  K72.91 Hepatic failure, unspecified with coma  K73 Chronic hepatitis, not elsewhere classified  K73.0 Chronic persistent hepatitis, not elsewhere classified  K73.1 Chronic lobular hepatitis, not elsewhere classified  K73.2 Chronic active hepatitis, not elsewhere classified  K73.8 Other chronic hepatitis, not elsewhere classified  K73.9 Chronic hepatitis, unspecified  K74 Fibrosis and cirrhosis of liver  K74.0 Hepatic fibrosis  K74.1 Hepatic sclerosis  K74.2 Hepatic fibrosis with hepatic sclerosis  K74.3 Primary biliary cirrhosis  K74.4 Secondary biliary cirrhosis  K74.5 Biliary cirrhosis, unspecified  K74.6 Other and unspecified cirrhosis of liver  K74.60 Unspecified cirrhosis of liver  K74.69 Other cirrhosis of liver  K75 Other inflammatory liver diseases  K75.0 Abscess of liver  K75.1 Phlebitis of portal vein  K75.2 Nonspecific reactive hepatitis  K75.3 Granulomatous hepatitis, not elsewhere classified  K75.4 Autoimmune hepatitis  K75.8 Other specified inflammatory liver diseases  K75.81 Nonalcoholic steatohepatitis (NASH)  K75.89 Other specified inflammatory liver diseases  K75.9 Inflammatory liver disease, unspecified  K76 Other diseases of liver  K76.0 Fatty (change of) liver, not elsewhere classified  K76.1 Chronic passive congestion of liver  K76.2 Central hemorrhagic necrosis of liver  K76.3 Infarction of liver  K76.4 Peliosis hepatis  K76.5 Hepatic veno-occlusive disease  K76.6 Portal hypertension  K76.7 Hepatorenal syndrome  K76.8 Other specified diseases of liver  K76.81 Hepatopulmonary syndrome  K76.89 Other specified diseases of liver  K76.9 Liver disease, unspecified  K77 Liver disorders in diseases classified elsewhere |
| Acute renal failure | N17.8 Other acute kidney failure  N17.9 Acute kidney failure, unspecified |
| Moderate to severe chronic renal failure | N18.3 Chronic kidney disease, stage 3 (moderate)  N18.4 Chronic kidney disease, stage 4 (severe) |
| hypothyroidism | E03.9 Hypothyroidism, unspecified |
| hyperparathyroidism | E21.3 Hyperparathyroidism, unspecified |
| hyperaldosteronism | E26 Hyperaldosteronism  E26.0 Primary hyperaldosteronism  E26.01 Conn's syndrome  E26.02 Glucocorticoid-remediable aldosteronism  E26.09 Other primary hyperaldosteronism  E26.1 Secondary hyperaldosteronism  E26.8 Other hyperaldosteronism  E26.81 Bartter's syndrome  E26.89 Other hyperaldosteronism  E26.9 Hyperaldosteronism, unspecified |
| hypercholesterolemia | E78.0 Pure hypercholesterolemia |
| Diabetes Mellitus | E10 Type 1 diabetes mellitus  E11 Type 2 diabetes mellitus  E13 Other specified diabetes mellitus |
| hypogonadism | Androgen deprivation therapy (ADT) due to prostate cancer,  E29.1 Testicular hypofunction |
| Subarachnoid hemorrhage | I60 Nontraumatic subarachnoid hemorrhage |
| intracranial trauma, | S06 Intracranial injury |

**Table S2. List of concerned co-medications**

| **High risk medications listed in CredibleMeds** |
| --- |
| Aclarubicin, Amiodarone, Anagrelide, Arsenic trioxide, Astemizole, Azithromycin, Bepridil, Cesium, Chloroquine, Chlorpromazine, Chlorprothixene, Cilostazol, Ciprofloxacin, Cisapride, Citalopram, Clarithromycin, Cocaine, Disopyramide, Dofetilide, Domperidone, Donepezil, Dronedarone, Droperidol, Erythromycin, Escitalopram, Flecainide, Fluconazole, Gatifloxacin, Grepafloxacin, Halofantrine, Haloperidol, Hydroquinidine, Hydroxychloroquine, Ibogaine, Ibutilide, Levofloxacin, Levomepromazine, Levomethadyl, Levosulpiride, Meglumine, Mesoridazine, Methadone, Moxifloxacin, Nifekalant, Ondansetron, Oxaliplatin, Papaverine, Pentamidine, Pimozide, Probucol, Procainamide, Propofol, Quinidine, Roxithromycin, Sertindole, Sevoflurane, Sotalol, Sparfloxacin, Sulpiride, Sultopride, Terfenadine, Terlipressin, Terodiline,Thioridazine, Vandetanib |
| **Possible risk medications listed in CredibleMeds** |
| Abarelix, Alfuzosin, Alimemazine, Apalutamide, Apomorphine, Aripiprazole, Coartem®(artemether / lumefantrine), Eurartesim®(artenimol / piperaquine), Asenapine, Atomoxetine, Bedaquiline, Bendamustine, Betrixaban, Bicalutamide, Bortezomib, Bosutinib, Buprenorphine, Cabozantinib, Capecitabine, Carbetocin, Ceritinib, Clofazimine, Clotiapine, Clozapine, Cobimetinib, Crizotinib, Cyamemazine, Dabrafenib, Dasatinib, Degarelix® Delamanid, Desipramine, Deutetrabenazine, Dexmedetomidine, Nuedexta (dextromethorphan / quinidine), Dolasetron, Efavirenz, Eliglustat, Encorafenib, Entrectinib, Epirubicin, Eribulin, Ezogabine, Felbamate, Fingolimod, Fluorouracil, Flupentixol, Gemifloxacin, Gilteritinib, Glasdegib, Granisetron, Hydrocodone, Iloperidone, Imipramine, Inotuzumab, Isradipine, Ivosidenib, Ketanserin, Lacidipine, Lapatinib, Lefamulin, Lenvatinib, Leuprolide, Levetiracetam, Levomethadone, Linezolid, Lithium, Lofexidine, Kaletra® (lopinavir / ritonavir), Lumateperone, Lurasidone, Maprotiline, Melperone, Mianserin, Midostaurin, Mifepristone, Mirabegron, Mirtazapine, Necitumumab, Nicardipine, Nilotinib, Norfloxacin, Nortriptyline, Nusinersen, Ofloxacin, Oliceridine, Osilodrostat, Osimertinib, Oxytocin, Ozanimod, Paliperidone, Palonosetron, Panobinostat, Pasireotide, Pazopanib, Perflutren, Perphenazine, Pilsicainide, Pimavanserin, Pipamperone, Pitolisant, Ponesimod, Pretomanid, Primaquine, Promethazine, Prothipendyl, Relugolix, Remimazolam, Ribociclib, Rilpivirine, Romidepsin, Rucaparib, Saquinavir, Selpercatinib, Siponimod, Sorafenib, Sunitinib, Tacrolimus, Tamoxifen, Tazemetostat, Telavancin, Telithromycin, Tetrabenazine, Tiapride Lonsurf® (tipiracil / trifluridine), Tizanidine, Tolterodine, Toremifene, Tramadol, Trimipramine, Tropisetron, Valbenazine, Vardenafil, Vemurafenib, Venlafaxine, Voclosporin, Vorinostat, Zotepine, Zuclopenthixol |
| **Concomitant use prescribed medications might cause hypokalemia** |
| Bumetanide, Digoxin, Furosemide, Mannitol, Trichlormethiazide |
| **Concomitant use prescribed medications might cause hyperkalemia** |
| Spironolactone, Eplerenone, Amtrel® (amlodipine / benazepril), Captopril, Imidapril, Ramipril, Entresto® (sacubitril / valsartan), Exforge® (amlodipine / valsartan), Twynsta® (amlodipine / telmisartan), Sevikar® (amlodipine / olmesartan medoxomil), Irbesartan Losartan, Telmisartan, Valsartan |
| **Concomitant use prescribed compound medications might cause abnormal potassium** |
| Amizide® (hydrochlorothiazide / amiloride), Coaprovel® (irbesartan / hydrochlorothiazide), Hyzaar® (losartan / hydrochlorothiazide), CO-Diovan® (valsartan / hydrochlorothiazide), Exforge HCT® (amlodipine / valsartan / hydrochlorothiazide), Sevikar HCT® (amlodipine / olmesartan / hydrochlorothiazide), Preterax® (perindopril / indapamide) |
| **Concomitant use prescribed medications as strong CYP3A4 inhibitor (listed based on medication category)** |
| Atazanavir, Dasabuvir, Ceritinib, Clarithromycin, Mifepristone, Kaletra®(ritonavir / lopinavir), Prezcobix®(darunavir / cobicistat), Symtuza®(darunavir / cobicistat), Viekirax® (Ombitasvir / paritaprevir / ritonavir), Itraconazole, Posaconazole, Voriconazole |


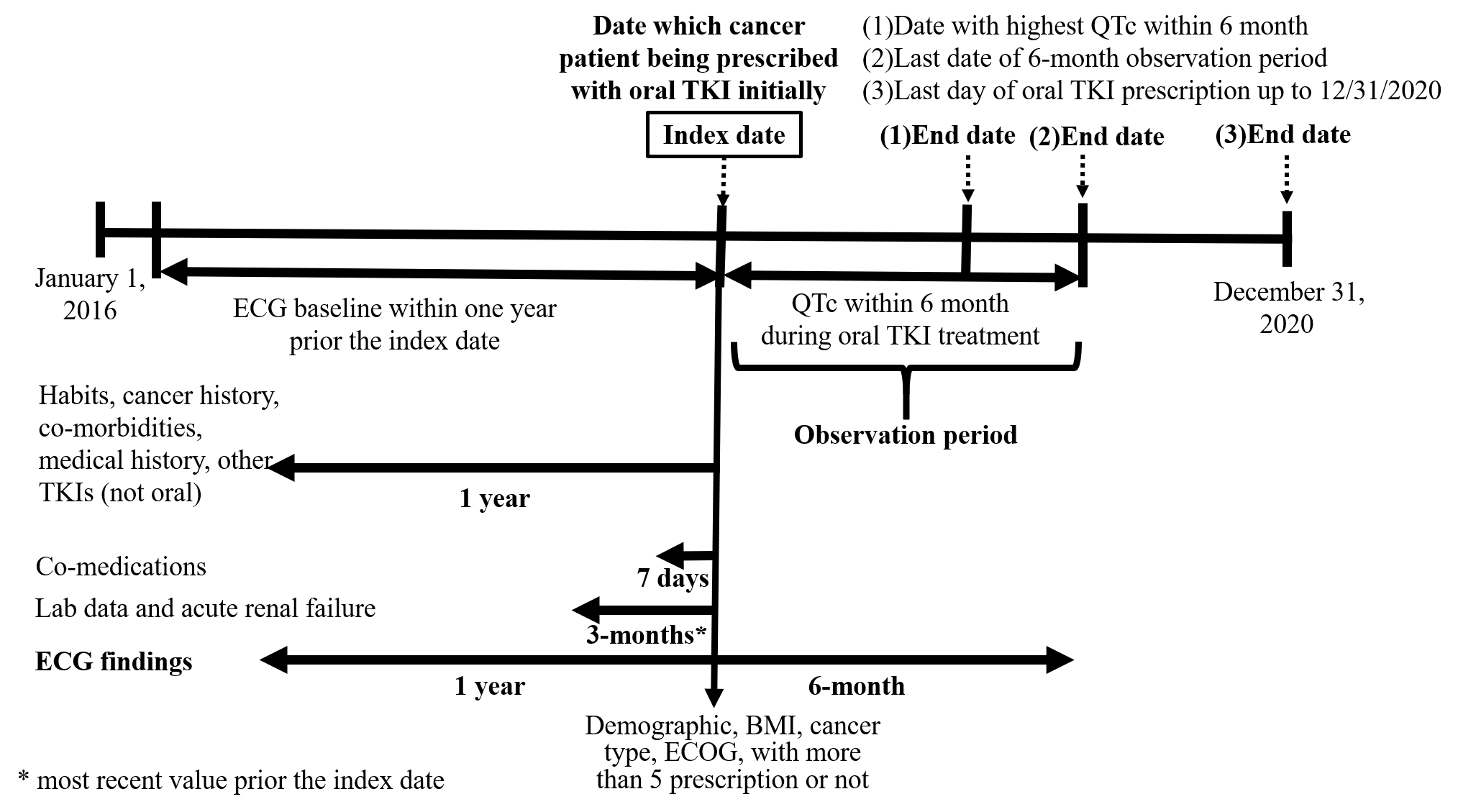


**Figure S1: Study design of retrospective cohort study**

TKI=tyrosine kinase inhibitor; QTc=Corrected QT Interval; ECG= electrocardiography; BMI= body mass index; ECOG= Eastern Cooperative Oncology Group Performance Status Scale;

**Table S3. Patients’ characteristics, co-morbidities, co-medications, laboratory data and ECG findings**

| **Variables/features** | **n** | **%, mean ± S.D.** |
| --- | --- | --- |
| **Total sample** | **400** |  |
| **Demographic and cancer status on the index date** |  |  |
| Male | 298 | 74.5 |
| Age | 400 | 63.5 ± 12.0 |
| Age ≥ 45 when initiating oral TKI | 372 | 93 |
| Cancer type |  |  |
| Solid tumor-liver cancer | 326 | 81.4 |
| Solid tumor-not liver cancer | 41 | 10.3 |
| Hematology cancer | 33 | 8.3 |
| Cancer stage |  |  |
| Solid tumor-stage I | 19 | 4.8 |
| Solid tumor-stage II | 47 | 11.8 |
| Solid tumor-stage III | 116 | 29.0 |
| Solid tumor-stage IV | 184 | 46.0 |
| Hematology cancer- Chronic phase | 13 | 3.3 |
| Hematology cancer- Accelerated phase | 4 | 1.0 |
| Hematology cancer- Blast phase | 4 | 1.0 |
| Hematology cancer- none differentiate phase | 13 | 3.3 |
| With metastasis when using TKI initially | 191 | 47.8 |
| With recurrent cancer when using TKI initially | 164 | 41.0 |
| ECOG-0 | 67 | 16.8 |
| ECOG-1 | 232 | 58.0 |
| ECOG-2 | 50 | 12.5 |
| ECOG-3 | 36 | 9.0 |
| ECOG-4 | 15 | 3.8 |
| BMI | 398 | 23.4 ± 4.1 |
| Smoking habit | 187 | 46.5 |
| Alcohol habit | 68 | 17.0 |
| **Usage of TKI for cancer treatment on the index date** | | |
| VEGFi of TKI-Sorafenib | 334 | 83.5 |
| VEGFi of TKI-Sunitinib | 28 | 7.0 |
| BCR-ABLi of TKI-Imatinib | 19 | 4.8 |
| BCR-ABLi of TKI-Nilotinib | 7 | 1.8 |
| BCR-ABLi of TKI-Dasatinib | 12 | 3.0 |
| Total DDD of the used TKI on index date | 400 | 0.92 ± 0.25 |
| Total DDD of the used TKI on the end date | 400 | 0.94 ± 0.23 |
| Days of using TKI | 400 | 65.7 ± 51.9 |
| Concurrent use with the other type of TKIs | 3 | 0.8 |
| Use the other TKI within one year after the oral TKI | 28 | 7.0 |
| **Co-morbidities or medical history (within one-year period prior index date)** | | |
| Ischemia heart disease | 32 | 8.0 |
| Cardiomyopathy | 5 | 1.3 |
| Heart failure | 34 | 8.5 |
| Heart surgery history prior index date | 3 | 0.8 |
| Arrythmia | 16 | 4.0 |
| Other CV disease ^#^ | 25 | 6.3 |
| With hypertension diagnosis prior index date | 156 | 39.0 |
| Diagnosed with hypertension on end day | 194 | 48.5 |
| Systolic blood pressure (mmHg) | 400 | 134.2 ± 21.8 |
| Diastolic blood pressure (mmHg) | 400 | 81.6 ± 13.8 |
| Diabetes mellitus | 137 | 34.3 |
| Endocrine disease | 44 | 11.0 |
| Neurological disease | 47 | 11.8 |
| Hepatitis C | 81 | 20.3 |
| Hepatitis B | 156 | 39.0 |
| Liver transplantation | 31 | 7.8 |
| Liver injury | 59 | 14.8 |
| Acute renal failure | 19 | 4.8 |
| Moderate to severe chronic kidney failure | 10 | 2.5 |
| **Co-medications within 7-day period prior the index date** |  |  |
| Concurrent use with strong CYP3A4 inhibitor | 1 | 0.3 |
| Concurrent use medications ≥ 5 items | 198 | 49.5 |
| Use high risk medication of CredibleMeds ≥1 item | 37 | 9.3 |
| Use possible risk CredibleMeds ≥1 item | 46 | 11.5 |
| Concomitant use Rx cause hypokalemia | 86 | 21.5 |
| Concomitant use Rx cause hyperkalemia | 69 | 17.3 |
| Concomitant use compound Rx cause abnormal potassium | 13 | 3.3 |
| **Lab data (most recent value within three month of the index date)** | | |
| AST (IU/L) | 384 | 96.9 ± 217.9 |
| ALT (IU/L) | 400 | 58.7 ± 100.1 |
| Total Bilirubin (mg/dL) | 389 | 1.6 ± 2.2 |
| INR values | 390 | 1.2 ± 0.3 |
| AFP (ng/mL) | 315 | 6595.1 ± 15016.2 |
| eGFR (mL/min) | 400 | 79.3 ± 34.3 |
| Hb (g/dL) | 400 | 11.9 ± 2.4 |
| LDH (U/L) | 99 | 394.4 ± 303.1 |
| CRP (mg/L) | 242 | 7.5 ± 7.4 |
| Albumin (g/dL) | 368 | 3.5 ± 0.7 |
| Potassium (mmol/L) | 379 | 4.0 ± 0.7 |
| Calcium (mmol/L) | 195 | 8.5 ± 0.8 |
| Magnesium (mmol/L) | 109 | 1.9 ± 0.3 |
| Sodium (mmol/L) | 382 | 134.5 ± 5.5 |
| **ECG findings in different time points** |  |  |
| QTc in baseline | 400 | 436.3 ± 29.7 |
| QTc prolongation in baseline | 93 | 23.3 |
| QTc by the end date | 400 | 452.7 ± 38.9 |
| QTc prolongation by the end date | 166 | 41.5 |
| ECG examination when medical visit |  |  |
| In outpatient units | 38 | 9.5 |
| In emergency room | 142 | 35.5 |
| In inpatient units | 220 | 55.0 |

CV=cardiovascular disease; ECG= electrocardiography; TKI=tyrosine kinase inhibitor; QTc=Corrected QT Interval; ECOG= Eastern Cooperative Oncology Group Performance Status Scale; BMI=body mass index; VEGFi= Vascular Endothelial Growth Factor inhibitor; DDD=defined daily dose (The assumed average maintenance dose per day for a drug used for its main indication in adults <https://www.who.int/tools/atc-ddd-toolkit/about-ddd> ); CYP3A4= cytochrome P450 3A4 family; Rx=prescribed medication; AST= aspartate aminotransferase; ALT= alanine aminotransferase ;INR= International Normalized Ratio; AFP= alpha-fetoprotein ; eGFR= estimated Glomerular filtration rate; Hb= hemoglobin; LDH= lactate dehydrogenase; CRP=C-reactive protein; HTN=hypertension;

#Other CV diseases= those CV diseases, excluding the stand-alone CV diseases which were strongly related to QTc prolongation (i.e., arrythmia [ICD 10 as I44 - I49], ischemia heart disease, hypertension, heart failure, cardiomyopathy), were grouped together to compare.

**Table S4. Comparisons of variables between that in training and testing dataset**

|  | **Training dataset** | | **Testing dataset** | | ***p*-value** |
| --- | --- | --- | --- | --- | --- |
|  | **n=279** | | **n=121** | |  |
| **Variables/features** | **n** | **(%)** | **n** | **(%)** |  |
| **Demographic and cancer status on the index date** |  |  |  |  |  |
| Male | 205 | (73.5) | 93 | (76.9) | 0.476 |
| Age ≥ 45 when initiate TKI | 260 | (93.2) | 112 | (92.6) | 0.821 |
| Hematology | 23 | (8.2) | 10 | (8.3) | 0.994 |
| With metastasis | 141 | (50.5) | 50 | (41.3) | 0.090 |
| Recurrent of cancer | 117 | (41.9) | 47 | (38.8) | 0.564 |
| ECOG score ≥ 3 | 38 | (13.6) | 13 | (10.7) | 0.428 |
| BMI ≥ 27 | 13 | (4.7) | 8 | (6.7) | 0.415 |
| Smoking habit | 127 | (45.5) | 60 | (49.6) | 0.454 |
| Alcohol habit | 49 | (17.6) | 19 | (15.7) | 0.649 |
| **Usage of TKI for cancer treatment on the index date** | | | | | |
| DDD ≥ 1 on index date | 220 | (78.9) | 98 | (81.0) | 0.626 |
| DDD ≥ 1 on the end date | 233 | (83.5) | 100 | (82.6) | 0.831 |
| Days of TKI ≥ 60 days | 133 | (47.7) | 50 | (41.3) | 0.242 |
| Use VEGFi of TKI | 251 | (90.0) | 111 | (91.7) | 0.579 |
| Ever used other TKI before | 21 | (7.5) | 7 | (5.8) | 0.531 |
| **ECG findings in different time points** |  |  |  |  |  |
| QTc prolongation in the baseline | 60 | (21.5) | 33 | (27.3) | 0.210 |
| Check ECG in emergency room | 100 | (35.8) | 42 | (34.7) | 0.828 |
| **Co-morbidities or medical history (within one-year period prior index date)** | | | | | |
| Ischemia heart disease | 23 | (8.2) | 9 | (7.4) | 0.785 |
| Cardiomyopathy | 3 | (1.1) | 2 | (1.7) | 0.641 |
| Heart failure | 24 | (8.6) | 10 | (8.3) | 0.911 |
| Heart surgery history prior index date | 2 | (0.7) | 1 | (0.8) | 1.000 |
| Arrythmia | 12 | (4.3) | 4 | (3.3) | 0.641 |
| Other CV diseases^#^ | 19 | (6.8) | 6 | (5.0) | 0.482 |
| With HTN diagnosis prior index date | 108 | (38.7) | 48 | (39.7) | 0.857 |
| Diagnosed with hypertension on end day | 132 | (47.3) | 62 | (51.2) | 0.470 |
| Systolic blood pressure ≥ 140 mmHg | 109 | (39.1) | 44 | (36.4) | 0.609 |
| Diastolic blood pressure ≥ 90 mmHg | 78 | (28.0) | 33 | (27.3) | 0.888 |
| Diabetes mellitus | 90 | (32.3) | 47 | (38.8) | 0.202 |
| Endocrine diseases | 36 | (12.9) | 8 | (6.6) | 0.065 |
| Neurological diseases | 37 | (13.3) | 10 | (8.3) | 0.154 |
| Hepatitis C | 56 | (20.1) | 25 | (20.7) | 0.893 |
| Hepatitis B | 114 | (40.9) | 42 | (34.7) | 0.247 |
| Liver transplantation | 21 | (7.5) | 10 | (8.3) | 0.800 |
| Liver damage (upon ICD9 or 10) | 36 | (12.9) | 23 | (19.0) | 0.114 |
| Acute renal failure | 7 | (5.8) | 12 | (4.3) | 0.522 |
| Moderate to severe chronic renal failure | 5 | (1.8) | 5 | (4.1) | 0.169 |
| **Co-medication within 7-day period prior the index date** |  |  |  |  |  |
| Concurrent use medications ≥ 5 items | 138 | (49.5) | 60 | (49.6) | 0.982 |
| Use high risk Rx of CredibleMeds ≥1 item | 25 | (9.0) | 12 | (9.9) | 0.762 |
| Use possible risk Rx of CredibleMeds ≥1 item | 31 | (11.1) | 15 | (12.4) | 0.711 |
| Concomitant use Rx cause hypokalemia | 62 | (22.2) | 24 | (19.8) | 0.593 |
| Concomitant use Rx cause hyperkalemia | 46 | (16.5) | 23 | (19.0) | 0.540 |
| Concomitant use compound Rx cause abnormal potassium | 10 | (3.6) | 3 | (2.5) | 0.567 |
| **Labe data** |  |  |  |  |  |
| AST ≥ 3 upper normal limits (120 IU/L) | 50 | (18.5) | 25 | (21.9) | 0.441 |
| ALT ≥ 3 upper normal limits (120 IU/L) | 22 | (7.9) | 6 | (5.0) | 0.292 |
| Total bilirubin > 2 mg/dL | 39 | (14.4) | 24 | (20.2) | 0.158 |
| INR > 1.5 | 14 | (5.1) | 7 | (6.0) | 0.711 |
| AFP ≥ 9 ng/mL | 154 | (70.6) | 74 | (76.3) | 0.301 |
| eGFR < 30 mL/min | 29 | (10.4) | 6 | (5.0) | 0.077 |
| Hb < 9 g/dL | 39 | (14.0) | 10 | (8.3) | 0.109 |
| LDH ≥ 225 U/L | 54 | (72.0) | 17 | (68.0) | 0.703 |
| CRP > 10 mg/L | 47 | (28.3) | 25 | (32.9) | 0.469 |
| Albumin < 3.8 g/dL | 160 | (63.0) | 63 | (55.3) | 0.161 |
| Potassium < 3.5 mmol/L | 45 | (17.1) | 27 | (23.3) | 0.158 |
| Calcium < 8 mmol/L | 34 | (24.8) | 15 | (25.9) | 0.878 |
| Magnesium < 1.7 mmol/L | 12 | (15.4) | 8 | (25.8) | 0.205 |
| Sodium < 135 mmol/L | 123 | (46.2) | 50 | (43.1) | 0.571 |

CV=cardiovascular disease; TKI=tyrosine kinase inhibitor; ECOG= Eastern Cooperative Oncology Group Performance Status Scale; BMI=body mass index; DDD=defined daily dose (The assumed average maintenance dose per day for a drug used for its main indication in adults <https://www.who.int/tools/atc-ddd-toolkit/about-ddd> ); VEGFi= Vascular Endothelial Growth Factor inhibitor; QTc= Corrected QT Interval; ECG= electrocardiography; HTN=hypertension; ICD= The International Statistical Classification of Diseases and Related Health Problems 9 or 10th Revision; Rx=prescribed medication; AST= aspartate aminotransferase; ALT= alanine aminotransferase; INR= International Normalized Ratio; AFP= alpha-fetoprotein; eGFR= estimated Glomerular filtration rate; Hb= hemoglobin; LDH= lactate dehydrogenase; CRP=C-reactive protein;

#Other CV diseases= those CV diseases, excluding the stand-alone CV diseases which were strongly related to QTc prolongation (i.e., arrythmia [ICD 10 as I44 - I49], ischemia heart disease, hypertension, heart failure, cardiomyopathy), were grouped together to compare.

**Table S5. Comparisons of continuous variables before and after imputation**

|  | | **Before imputation** | | **After imputation for missing data** | |  |
| --- | --- | --- | --- | --- | --- | --- |
| **Name of parameter** | | **n** | **Mean ± S.D.** | **n** | **Mean ± S.D.** | ***p*-value** |
| **Training dataset n=279** | |  |  |  |  |  |
|  | AST (IU/L) | 270 | 98.60 ± 246.78 | 9 | 102.62 ± 31.38 | 0.96 |
|  | AFP (ng/mL) | 218 | 5889.08 ± 14097.97 | 61 | 6165.94 ± 1514.98 | 0.78 |
|  | LDH (U/L) | 75 | 488.53 ± 840.80 | 204 | 518.88 ± 103.06 | 0.76 |
|  | CRP (mg/L) | 166 | 7.63 ± 7.42 | 113 | 7.32 ± 0.81 | 0.60 |
|  | Total bilirubin (mg/dL) | 270 | 1.53 ± 2.26 | 9 | 1.47 ± 0.37 | 0.94 |
|  | INR | 274 | 1.15 ± 0.27 | 5 | 1.11 ± 0.05 | 0.77 |
|  | Albumin (g/dL) | 254 | 3.43 ± 0.72 | 25 | 3.41 ± 0.28 | 0.79 |
|  | Potassium (mmol/L) | 263 | 4.07 ± 0.78 | 16 | 4.09 ± 0.08 | 0.77 |
|  | Calcium (mmol/L) | 137 | 8.48 ± 0.78 | 142 | 8.56 ± 0.19 | 0.27 |
|  | Sodium (mmol/L) | 266 | 134.43 ± 5.70 | 13 | 134.77 ± 0.68 | 0.83 |
| **Testing dataset n=121** | |  |  |  |  |  |
|  | AST (IU/L) | 114 | 92.73 ± 126.31 | 7 | 110.67 ± 44.15 | 0.71 |
|  | AFP (ng/mL) | 119 | 1.78 ± 2.14 | 2 | 2.39 ± 0.50 | 0.69 |
|  | LDH (U/L) | 116 | 1.16 ± 0.24 | 5 | 1.23 ± 0.11 | 0.50 |
|  | CRP (mg/L) | 97 | 8181.78 ± 16869.76 | 24 | 8891.33 ± 2588.01 | 0.69 |
|  | Total bilirubin (mg/dL) | 25 | 381.88 ± 251.76 | 96 | 382.86 ± 77.77 | 0.99 |
|  | INR | 76 | 7.24 ± 7.25 | 45 | 7.41 ± 0.96 | 0.85 |
|  | Albumin (g/dL) | 114 | 3.54 ± 0.70 | 7 | 3.44 ± 0.23 | 0.71 |
|  | Potassium (mmol/L) | 116 | 3.87 ± 0.65 | 5 | 3.94 ± 0.01 | 0.83 |
|  | Calcium (mmol/L) | 58 | 8.41 ± 0.79 | 63 | 8.46 ± 0.23 | 0.62 |
|  | Sodium (mmol/L) | 116 | 134.72 ± 4.95 | 5 | 134.81 ± 0.02 | 0.86 |

AST= aspartate aminotransferase; ALT= alanine aminotransferase; INR= International Normalized Ratio; AFP= alpha-fetoprotein; eGFR= estimated Glomerular filtration rate; Hb= hemoglobin; LDH= lactate dehydrogenase; CRP=C-reactive protein;

**Table S6. Sorting the variables of interest based on the odds ratio for the data in the training dataset before imputation.**

| **Variable** | **OR*** | **95% CI** | | **P-value** |
| --- | --- | --- | --- | --- |
| QTc prolongation in the baseline | 14.88 | 6.52 | -33.93 | <0.001* |
| Total bilirubin > 2 mg/dL | 5.149 | 2.258 | -11.743 | <0.001* |
| Calcium < 8 mmol/L | 5.222 | 1.374 | -19.843 | 0.015* |
| Potassium < 3.5 mmol/L | 2.530 | 1.298 | -4.933 | 0.006* |
| Albumin < 3.8 g/dL | 2.758 | 1.579 | -4.815 | <0.001* |
| AST ≥ 3 upper normal limits (105 IU/L) | 5.221 | 2.635 | -10.345 | <0.001* |
| Check ECG in emergency room | 2.400 | 1.369 | -4.207 | 0.002* |
| ECOG score ≥ 3 | 3.883 | 1.419 | -10.626 | 0.008* |
| INR > 1.5 | 1.207 | 0.264 | -5.522 | 0.808 |
| Other CV diseases^#^ | 9.886 | 2.137 | -45.726 | 0.003* |
| Concurrent use medications ≥ 5 items | 2.055 | 1.201 | -3.517 | 0.009* |
| Arrythmia | 10.214 | 1.209 | -86.303 | 0.033* |
| Male | 1.539 | 0.808 | -2.934 | 0.190 |
| Concomitant use Rx cause hypokalemia | 3.377 | 1.639 | -6.955 | 0.001* |
| Use high risk Rx of CredibleMeds ≥1 item | 8.658 | 2.413 | -31.070 | 0.001* |
| Sodium < 135 mmol/L | 1.849 | 1.085 | -3.153 | 0.024* |
| BMI ≥ 27 | 1.157 | 0.614 | -2.177 | 0.652 |
| Neurological diseases | 1.675 | 0.668 | -4.200 | 0.271 |
| Ischemia heart disease | 2.126 | 0.806 | -5.605 | 0.128 |
| Age ≥ 45 when initiate TKI | 2.134 | 0.673 | -6.760 | 0.198 |
| Recurrent of cancer | 0.622 | 0.366 | -1.057 | 0.080 |
| Concomitant use Rx cause hyperkalemia | 1.868 | 0.906 | -3.849 | 0.090 |
| CRP > 10 mg/L | 1.740 | 0.837 | -3.615 | 0.138 |
| DDD ≥ 1 on index date | 0.661 | 0.318 | -1.374 | 0.267 |
| AFP ≥ 9 ng/mL | 0.628 | 0.338 | -1.165 | 0.140 |
| LDH ≥ 225 U/L | 2.933 | 0.619 | -13.897 | 0.175 |
| Hb < 9 g/dL | 1.875 | 0.731 | -4.810 | 0.191 |
| Smoking habit | 1.537 | 0.905 | -2.610 | 0.112 |
| With metastasis | 1.361 | 0.802 | -2.310 | 0.254 |
| Use possible risk Rx of CredibleMeds ≥1 item | 0.729 | 0.301 | -1.766 | 0.484 |
| DDD ≥ 1 on the end date | 0.806 | 0.389 | -1.671 | 0.563 |
| Moderate to severe chronic renal failure | 1.614 | 0.223 | -11.663 | 0.635 |
| Diagnosed with hypertension on end day | 1.801 | 1.058 | -3.066 | 0.030 |
| Hematology | 1.434 | 0.501 | -4.099 | 0.501 |
| Alcohol habit | 1.408 | 0.717 | -2.764 | 0.320 |
| With HTN diagnosis history prior index date | 1.578 | 0.917 | -2.717 | 0.100 |
| Days of TKI ≥ 60 days | 0.819 | 0.483 | -1.387 | 0.457 |
| Heart failure | 1.909 | 0.688 | -5.460 | 0.288 |
| Liver transplantation | 0.500 | 0.191 | -1.311 | 0.159 |
| eGFR < 30 mL/min | 0.698 | 0.208 | -2.336 | 0.559 |
| Endocrine diseases | 1.180 | 0.456 | -3.054 | 0.734 |
| Liver damage (upon ICD9 or 10) | 1.711 | 0.774 | -3.780 | 0.185 |
| Systolic blood pressure ≥ 140 mmHg | 0.757 | 0.438 | -1.308 | 0.319 |
| Diabetes mellitus | 1.840 | 1.046 | -3.238 | 0.034 |
| Hepatitis B | 0.822 | 0.485 | -1.396 | 0.469 |
| Use VEGFi of TKI | 0.603 | 0.218 | -1.668 | 0.330 |
| Heart surgery history prior index date | - | - |  | - |
| Diastolic blood pressure ≥ 90 mmHg | 0.874 | 0.489 | -1.564 | 0.650 |
| Cardiomyopathy | 1.614 | 0.223 | -11.563 | 0.635 |
| Ever used other TKI before | 0.882 | 0.286 | -2.722 | 0.828 |
| Magnesium < 1.7 mmol/L | 1.628 | 0.397 | -6.679 | 0.499 |
| ALT ≥ 3 upper normal limits (120 IU/L) | 0.882 | 0.286 | -2.722 | 0.828 |
| Hepatitis C | 0.961 | 0.511 | -1.806 | 0.902 |
| Concomitant use compound Rx cause abnormal potassium | 0.795 | 0.143 | -4.434 | 0.794 |
| Acute renal failure | 10.214 | 1.209 | -86.303 | 0.033 |

CV=cardiovascular disease; OR=odds ratio; QTc= Corrected QT Interval; AST= aspartate aminotransferase; ECG= electrocardiography; ECOG= Eastern Cooperative Oncology Group Performance Status Scale; INR= International Normalized Ratio; BMI=body mass index; TKI=tyrosine kinase inhibitor; CRP=C-reactive protein; D DDD=defined daily dose (The assumed average maintenance dose per day for a drug used for its main indication in adults <https://www.who.int/tools/atc-ddd-toolkit/about-ddd> ); AFP= alpha-fetoprotein; LDH= lactate dehydrogenase; Hb= hemoglobin; Rx=prescribed medication; HTN=hypertension; eGFR= estimated Glomerular filtration rate; ICD= The International Statistical Classification of Diseases and Related Health Problems 9 or 10th Revision; VEGFi= Vascular Endothelial Growth Factor inhibitor; ALT= alanine aminotransferase;

#Other CV diseases= those CV diseases, excluding the stand-alone CV diseases which were strongly related to QTc prolongation (i.e., arrythmia [ICD 10 as I44 - I49], ischemia heart disease, hypertension, heart failure, cardiomyopathy), were grouped together to compare.

* the OR was obtained from the univariate logistic regression for each variable to explore the associated factors of QTc prolongation

- only one QTc prolongation event to not to perform the analysis

**Table S7. Sorting the variables of interest based on the odds ratio and *p* value for the data in the training dataset (from the most to lest statistically significant) after imputation**

| **Name of eligible parameter** | **OR** | **95% CI** | | ***p*-value** |
| --- | --- | --- | --- | --- |
| Prolongation of QTc in the baseline | 7.785 | 4.004 | -15.134 | < 0.001* |
| Total bilirubin > 2 mg/dL | 3.365 | 1.668 | -6.787 | 0.001* |
| Calcium < 8 mmol/L | 3.586 | 1.709 | -7.523 | 0.001* |
| Potassium < 3.5 mmol/L | 2.996 | 1.550 | -5.794 | 0.001* |
| Albumin < 3.8 g/dL | 2.398 | 1.396 | -4.119 | 0.002* |
| AST ≥ 3 upper normal limits (105 IU/L) | 2.443 | 1.335 | -4.471 | 0.004* |
| Check ECG in emergency room | 2.064 | 1.252 | -3.404 | 0.004* |
| ECOG score ≥ 3 | 2.666 | 1.322 | -5.376 | 0.006* |
| INR > 1.5 | 6.050 | 1.648 | -22.211 | 0.007* |
| Other CV diseases ^#^ | 3.582 | 1.318 | -9.729 | 0.012* |
| Concurrent use medications ≥ 5 items | 1.839 | 1.132 | -2.988 | 0.014* |
| Arrythmia | 4.853 | 1.284 | -18.345 | 0.020* |
| Male gender | 1.975 | 1.111 | -3.513 | 0.021* |
| Concomitant use RX cause hypokalemia | 1.863 | 1.054 | -3.294 | 0.032* |
| Use high risk Rx of CredibleMeds ≥1 item | 2.469 | 1.066 | -5.715 | 0.035* |
| Sodium < 135 mmol/L | 1.667 | 1.028 | -2.702 | 0.038* |
| BMI ≥ 27 | 1.833 | 0.980 | -3.427 | 0.058 |
| Neurological disease(s) | 1.952 | 0.972 | -3.919 | 0.060 |
| Ischemia heart disease | 2.096 | 0.885 | -4.963 | 0.092 |
| Age ≥ 45 when initiated TKI | 2.623 | 0.847 | -8.121 | 0.095 |
| Recurrent of cancer | 0.666 | 0.407 | -1.089 | 0.105 |
| Concomitant use Rx cause hyperkalemia | 1.648 | 0.873 | -3.112 | 0.124 |
| CRP > 10 mg/L | 1.568 | 0.835 | -2.946 | 0.162 |
| DDD of TKI on index date ≥ 1 | 0.671 | 0.376 | -1.197 | 0.177 |
| AFP ≥ 9 ng/mL | 0.685 | 0.39 | -1.203 | 0.188 |
| LDH ≥ 225 U/L | 0.578 | 0.246 | -1.360 | 0.209 |
| Hb < 9 g/dL | 1.528 | 0.774 | -3.016 | 0.221 |
| Smoking habit | 1.310 | 0.809 | -2.119 | 0.272 |
| With metastasis | 1.264 | 0.781 | -2.043 | 0.340 |
| Use possible risk CredibleMeds ≥1 item | 0.693 | 0.313 | -1.534 | 0.366 |
| DDD of TKI on end date ≥ 1 | 0.749 | 0.396 | -1.417 | 0.375 |
| Moderate & severe chronic kidney disease | 0.373 | 0.041 | -3.379 | 0.380 |
| Diagnosed with hypertension on end day | 1.233 | 0.762 | -1.993 | 0.394 |
| Hematology cancer | 1.430 | 0.608 | -3.365 | 0.413 |
| Alcohol habit | 1.291 | 0.693 | -2.405 | 0.421 |
| With HTN diagnosis prior index date | 1.210 | 0.741 | -1.976 | 0.447 |
| Days of using the TKI ≥ 60 days | 0.839 | 0.519 | -1.358 | 0.476 |
| Heart failure | 1.312 | 0.565 | -3.042 | 0.528 |
| Liver transplantation | 0.740 | 0.289 | -1.897 | 0.531 |
| eGFR < 30 mL/min | 0.776 | 0.347 | -1.739 | 0.539 |
| Endocrine diseases | 1.246 | 0.615 | -2.525 | 0.541 |
| Liver damage (upon ICD9 or 10) | 1.246 | 0.615 | -2.525 | 0.541 |
| Systolic blood pressure ≥ 140 mmHg | 0.861 | 0.526 | -1.411 | 0.553 |
| Diabetes mellitus | 1.161 | 0.697 | -1.935 | 0.566 |
| Hepatitis B | 1.107 | 0.68 | -1.802 | 0.682 |
| Use VEGF*i* | 0.868 | 0.394 | -1.914 | 0.726 |
| Heart surgery history prior index date | 1.518 | 0.094 | -24.526 | 0.769 |
| Diastolic blood pressure ≥ 90 mmHg | 0.926 | 0.542 | -1.583 | 0.778 |
| Cardiomyopathy | 0.755 | 0.068 | -8.422 | 0.819 |
| Ever used the other TKI before | 0.926 | 0.371 | -2.313 | 0.869 |
| Magnesium < 1.7 mmol/L | 1.085 | 0.336 | -3.508 | 0.892 |
| ALT ≥ 3 upper normal limits (120 IU/L) | 1.052 | 0.434 | -2.551 | 0.911 |
| Hepatitis C | 0.974 | 0.535 | -1.774 | 0.932 |
| Concomitant use compound Rx cause abnormal potassium | 1.009 | 0.278 | -3.661 | 0.989 |
| Acute renal failure | 3.184 | 0.935 | -10.843 | 0.999 |

CV=cardiovascular disease; OR=odds ratio; QTc= Corrected QT Interval; AST= aspartate aminotransferase; ECG= electrocardiography; ECOG= Eastern Cooperative Oncology Group Performance Status Scale; INR= International Normalized Ratio; BMI=body mass index; TKI=tyrosine kinase inhibitor; CRP=C-reactive protein; DDD=defined daily dose (The assumed average maintenance dose per day for a drug used for its main indication in adults <https://www.who.int/tools/atc-ddd-toolkit/about-ddd> ); AFP= alpha-fetoprotein; LDH= lactate dehydrogenase; Hb= hemoglobin; Rx=prescribed medication; HTN=hypertension; eGFR= estimated Glomerular filtration rate; ICD= The International Statistical Classification of Diseases and Related Health Problems 9 or 10th Revision; VEGFi= Vascular Endothelial Growth Factor inhibitor; ALT= alanine aminotransferase;

#Other CV diseases= those CV diseases, excluding the stand-alone CV diseases which were strongly related to QTc prolongation (i.e., arrythmia [ICD 10 as I44 - I49], ischemia heart disease, hypertension, heart failure, cardiomyopathy), were grouped together to compare.

*the order of variable elimination performed in standard backpropagation algorithm of artificial neural network (ANN) is the same as that performed of backward elimination logistic regression (LR).

**Table S8. Univariate logistic regression analysis among cancer patients with hepatocellular carcinoma or not in the training dataset**

|  | **HCC** | | | **Not HCC** | | |
| --- | --- | --- | --- | --- | --- | --- |
| **Variable** | **OR*** | **95% CI** | | **OR*** | **95% CI** | |
| QTc prolongation in the baseline | 10.62 | 4.80 | -23.51* | 2.90 | 0.78 | -10.78 |
| Total bilirubin > 2 mg/dL | 3.20 | 1.52 | -6.72* | 5.50 | 0.53 | -56.81 |
| Calcium < 8 mmol/L | 7.57 | 2.45 | -23.34* | 1.63 | 0.49 | -5.42 |
| Potassium < 3.5 mmol/L | 3.84 | 1.76 | -8.35* | 1.46 | 0.38 | -5.55 |
| Albumin < 3.8 g/dL | 2.92 | 1.61 | -5.28* | 0.91 | 0.22 | -3.70 |
| AST ≥ 3 upper normal limits (105 IU/L) | 2.30 | 1.21 | -4.36* | 5.50 | 0.53 | -56.81 |
| Check ECG in emergency room | 2.35 | 1.33 | -4.16* | 1.39 | 0.47 | -4.15 |
| ECOG score ≥ 3 | 3.32 | 1.46 | -7.53* | 1.37 | 0.32 | -5.79 |
| INR > 1.5 | 7.33 | 1.55 | -34.79* | 3.47 | 0.30 | -40.90 |
| Other CV diseases^#^ | 2.67 | 0.93 | -7.62 | - | - | - |
| Concurrent use medications ≥ 5 items | 1.97 | 1.14 | -3.38* | 1.44 | 0.48 | -4.38 |
| Arrythmia | 3.12 | 0.76 | -12.81 | - | - | - |
| Male | 1.91 | 0.98 | -3.73 | 2.22 | 0.70 | -7.10 |
| Concomitant use Rx cause hypokalemia | 2.19 | 1.13 | -4.25* | 1.20 | 0.37 | -3.86 |
| Use high risk Rx of CredibleMeds ≥1 item | 3.59 | 1.20 | -10.72* | 1.37 | 0.32 | -5.79 |
| Sodium < 135 mmol/L | 1.91 | 1.11 | -3.28* | 0.98 | 0.33 | -2.91 |
| BMI ≥ 27 | 1.84 | 0.93 | -3.67 | 1.77 | 0.39 | -7.97 |
| Neurological diseases | 3.23 | 1.37 | -7.63 | 0.54 | 1.26 | -2.33 |
| Ischemia heart disease | 2.27 | 0.83 | -6.20 | 1.72 | 0.31 | -9.45 |
| Age ≥ 45 when initiate TKI | 2.34 | 0.63 | -8.74 | 3.45 | 0.37 | -31.79 |
| Recurrent of cancer | 0.60 | 0.35 | -1.03 | 0.97 | 0.21 | -4.54 |
| Concomitant use Rx cause hyperkalemia | 1.73 | 0.85 | -3.51 | 1.37 | 0.32 | -5.79 |
| CRP > 10 mg/L | 1.63 | 0.82 | -3.24 | 1.25 | 0.25 | -6.24 |
| DDD ≥ 1 on index date | 0.61 | 0.31 | -1.22 | 0.78 | 0.25 | -2.42 |
| AFP ≥ 9 ng/mL | 0.61 | 0.33 | -1.11 | 2.67 | 0.28 | -25.64 |
| LDH ≥ 225 U/L | 0.27 | 0.68 | -1.08 | 0.99 | 0.27 | -3.54 |
| Hb < 9 g/dL | 1.19 | 0.52 | -2.76 | 2.87 | 0.83 | -9.99 |
| Smoking habit | 1.08 | 0.63 | -1.84 | 3.06 | 0.97 | -9.61 |
| With metastasis | 1.64 | 0.96 | -2.81 | 0.43 | 0.14 | -1.31 |
| Use possible risk Rx of CredibleMeds ≥1 item | 0.72 | 0.29 | -1.76 | 0.61 | 0.11 | -3.47 |
| DDD ≥ 1 on the end date | 0.88 | 0.40 | -1.96 | 0.48 | 0.15 | -1.52 |
| Moderate to severe chronic renal failure | 0.49 | 0.50 | -4.79 | - | - | - |
| Diagnosed with hypertension on end day | 1.77 | 1.03 | -3.04 | 0.27 | 0.09 | -0.86 |
| Hematology | - | - | - | 2.02 | 0.67 | -6.11 |
| Alcohol habit | 1.27 | 0.64 | -2.54 | 1.37 | 0.32 | -5.79 |
| With HTN diagnosis history prior index date | 1.50 | 0.86 | -2.60 | 0.55 | 0.18 | -1.66 |
| Days of TKI ≥ 60 days | 0.77 | 0.45 | -1.31 | 1.20 | 0.37 | -3.85 |
| Heart failure | 1.26 | 0.37 | -4.24 | 1.54 | 0.44 | -5.44 |
| Liver transplantation | 0.72 | 0.28 | -1.87 | - | - | - |
| eGFR < 30 mL/min | 0.89 | 0.32 | -2.53 | 0.65 | 0.17 | -2.47 |
| Endocrine diseases | 1.56 | 0.64 | -3.76 | 0.87 | 0.25 | -3.06 |
| Liver damage (upon ICD9 or 10) | 1.39 | 0.67 | -2.90 | - | - | - |
| Systolic blood pressure ≥ 140 mmHg | 0.78 | 2.45 | -1.35 | 1.29 | 0.41 | -4.01 |
| Diabetes mellitus | 1.30 | 0.73 | -2.29 | 0.73 | 0.23 | -2.39 |
| Hepatitis B | 1.20 | 0.70 | -2.04 | - | - | - |
| Use VEGFi of TKI | - | - | - | 0.67 | 0.22 | -1.99 |
| Heart surgery history prior index date | - | - | - | 1.49 | 0.09 | -24.20 |
| Diastolic blood pressure ≥ 90 mmHg | 0.94 | 0.53 | -1.68 | 0.78 | 0.17 | -3.51 |
| Cardiomyopathy | 0.80 | 0.07 | -9.41 | - | - | - |
| Ever used other TKI before | 1.50 | 0.21 | -10.85 | 0.84 | 0.26 | -2.75 |
| Magnesium < 1.7 mmol/L | 0.89 | 0.21 | -3.82 | 1.68 | 0.22 | -12.96 |
| ALT ≥ 3 upper normal limits (120 IU/L) | - | - | - | 1.03 | 0.42 | -2.53 |
| Hepatitis C | 0.93 | 0.50 | -1.74 | 1.65 | 0.10 | -27.87 |
| Concomitant use compound Rx cause abnormal potassium | 0.59 | 0.11 | -3.09 | 3.47 | 0.30 | -40.9 |
| Acute renal failure | 5.56 | 1.13 | -27.4 | 0.80 | 0.07 | -9.41 |

HCC= hepatocellular carcinoma; CV=cardiovascular disease; OR=odds ratio; QTc= Corrected QT Interval; AST= aspartate aminotransferase; ECG= electrocardiography; ECOG= Eastern Cooperative Oncology Group Performance Status Scale; INR= International Normalized Ratio; BMI=body mass index; TKI=tyrosine kinase inhibitor; CRP=C-reactive protein; DDD=defined daily dose (The assumed average maintenance dose per day for a drug used for its main indication in adults <https://www.who.int/tools/atc-ddd-toolkit/about-ddd> ); AFP= alpha-fetoprotein; LDH= lactate dehydrogenase; Hb= hemoglobin; Rx=prescribed medication; HTN=hypertension; eGFR= estimated Glomerular filtration rate; ICD= The International Statistical Classification of Diseases and Related Health Problems 9 or 10th Revision; VEGFi= Vascular Endothelial Growth Factor inhibitor; ALT= alanine aminotransferase;

#Other CV diseases= those CV diseases, excluding the stand-alone CV diseases which were strongly related to QTc prolongation (i.e., arrythmia [ICD 10 as I44 - I49], ischemia heart disease, hypertension, heart failure, cardiomyopathy), were grouped together to compare.

* the OR was obtained from the univariate logistic regression for each variable to explore the associated factors of QTc prolongation

- only one QTc prolongation event to not to perform the analysis

| **Table S9. 12 variables identified as the most important features through several iterations of feature selection using forward-swap and stepwise regression supervised learning using training dataset in SAS Viya** | | | | | |  |
| --- | --- | --- | --- | --- | --- | --- |
| **Importance** | **Selected feature** | **MSE** | **AIC** | **AICc** | **BIC** | |
| 1 | Prolongation of QTc in the baseline | 0.00304 | ✓ | ✓ | ✓ | |
| 2 | Calcium < 8 mmol/L | 0.00292 | ✓ | ✓ | ✓ | |
| 3 | Male gender | 0.00285 | ✓ | ✓ | ✓ | |
| 4 | Other CV diseases# | 0.00278 | ✓ | ✓ | ✓ | |
| 5 | Check ECG in emergency room | 0.00272 | ✓ | ✓ | ✓ | |
| 6 | AST ≥ 105 IU/L | 0.00268 | ✓ | ✓ | ✓ | |
| 7 | Potassium < 3.5 mmol/L | 0.00265 | ✓ | ✓ | ✓ | |
| 8 | DDD of TKI on index date ≥ 1 | 0.00262 | ✓ | ✓ | ✓ | |
| 9 | Concomitant use Rx cause hyperkalemia | 0.00259 | ✓ | ✓ |  | |
| 10 | AFP ≥ 9 ng/mL | 0.00257 | ✓ | ✓ |  | |
| 11 | Albumin < 3.8 g/dL | 0.00256 | ✓ |  |  | |
| 12 | Cardiomyopathy | 0.00254 | ✓ |  |  | |

1=feature contributes significantly to prediction accuracy (most important feature) to 12= as the least important feature. The lower value of MSE indicates that the model’s predictors are closer to the true values, reflecting better overall performance, while including the corresponding feature into the model and meeting the selection and stop criterion.

CV=cardiovascular disease; QTc= Corrected QT Interval; ECG= electrocardiography; AST= aspartate aminotransferase; DDD=defined daily dose (The assumed average maintenance dose per day for a drug used for its main indication in adults <https://www.who.int/tools/atc-ddd-toolkit/about-ddd> ); Rx=prescribed medication; AFP= alpha-fetoprotein; AIC= Akaike information criterion; AICc= Akaike information criterion corrected for small sample; BIC=Bayesian Information Criterion; MSE= mean squared error

√ means: parameters were selected based on the findings of AIC, AICc or BIC. The most important 12 parameters were selected based on the least AIC, 10 and 8 parameters were selected based on AICc or BIC, respectively.

#Other CV diseases= those CV diseases, excluding the stand-alone CV diseases which were strongly related to QTc prolongation (i.e., arrythmia [ICD 10 as I44 - I49], ischemia heart disease, hypertension, heart failure, cardiomyopathy), were grouped together to compare.

| **Table S10.** **Optimization numerical methods and hyperparameter settings of artificial neural network in SPSS or SAS Viya.** | | |
| --- | --- | --- |
| **ANN setting** | **SPSS** | **SAS Viya** |
| Hidden layer | 1 | 1 |
| Hidden layer startup function | Hyperbolic tangent | Hyperbolic tangent |
| Output layer startup function | Hyperbolic tangent | Hyperbolic tangent |
| Number of neurons | Automatic (1-50) | 20 |
| Optimization numerical methods | SCG | LBFGS |
| Other hyperparameter settings | Sampling: batch  Lambdainitial= 0.000005  Sigmainitial =0.00005  Intervaloffset=0.5  Memsize=1000 | Parameter estimation: iterative update  Target error function: normal  L1 weighted recession: 0.01  L2 weighted recession: 0.01  Execution stopped early: iteration stalled 5 times |

SCG: scaled conjugate gradient; LBFGS: limited-memory Broyden-Fltcher-Goldfarb-Shanno; ANN= artificial neural network

**Table S11.** **Model fitting criteria, risk coefficient, risk probability after controlling for other factors, cut-off point of predicted risk probability of QTc prolongation in training dataset for the 7 parameters commonly identified in both best model**

| **7-parameter model** | | | |
| --- | --- | --- | --- |
| **Model Fitting Criteria** | | |  |
|  | AIC | 110.376 |  |
|  | BIC | 139.425 |  |
|  | -2 Log Likelihood | 94.376 |  |
| **Variable (X_i_)** | **Variable definition** | **Coefficient (β_i_)** | **Risk Probability^1^ (ranking)** |
| 1 | **Intercept** | **-1.910** | **0.148** |
| 2 | Prolongation of QTc in the baseline | 1.986 | 0.519 (1) |
| 3 | Potassium < 3.5 mmol/L | 0.725 | 0.234 (5) |
| 4 | Calcium < 8 mmol/L | 1.053 | 0.298 (3) |
| 5 | Albumin < 3.8 g/dL | 0.502 | 0.197 (6) |
| 6 | AST ≥ 105 IU/L | 0.779 | 0.244 (4) |
| 7 | Check ECG in emergency room | 0.490 | 0.195 (7) |
| 8 | Other CV diseases^#^ | 1.552 | 0.411 (2) |
| 9 | Total bilirubin > 2 mg/dL | - |  |
| 10 | ECOG score ≥ 3 | - |  |
| 11 | Concurrent use medications ≥ 5 items | - |  |
| 12 | INR > 1.5 | - |  |
| **Cut-off point of predicted risk probability^+^** | | **0.459** |  |
|  | AUROC  (95 % CI) | 0.791  (0.736-0.846) |  |
|  | Sensitivity | 0.667 |  |
|  | Specificity | 0.815 |  |
|  | Youden index | 0.482 |  |

CV=cardiovascular diease; QTc= Corrected QT Interval; ECOG= Eastern Cooperative Oncology Group Performance Status Scale; ECG= electrocardiography; AST= aspartate aminotransferase; DDD=defined daily dose; TKI=tyrosine kinase inhibitor; Rx=prescribed medication; AFP= alpha-fetoprotein; AUROC= Area Under Receiver Operating Characteristics Curve; CI=confidence interval.

#Other CV diseases= those CV diseases, excluding the stand-alone CV diseases which were strongly related to QTc prolongation (i.e., arrythmia [ICD 10 as I44 - I49], ischemia heart disease, hypertension, heart failure, cardiomyopathy), were grouped together to compare.

^1^ Risk probability of QTc prolongation within 6 month among cancer patients newly treated with oral selected TKIs = exp^z^ /(1+exp^z^), where z=intercept +β_1_*X_1_**+** β_2_*X_2_**+** β_3_*X_3_**+** β_4_*X_4_**+** β_5_*X_5_**+** β_6_*X_6_**+**……+ β_i_*X*_i_*,(*i*= number of parameters being identified in the final best model), where exp refers exponential function.

^+^112 patients in training dataset (40.1%) had a predicted probability of ≥0.414

**Table S12. Prediction performance of the best selected models from different model selection and training approaches in training and testing datasets and in different subgroups analyses**

|  | **Cut-off point** | **AUROC** | **95% CI** | **Sen.** | **Spe.** | **Youd-en index** | **% of patients with predicted probability > cut-off point**  **(positive)** |
| --- | --- | --- | --- | --- | --- | --- | --- |
| **Statistical 12-parameter model (the best model)** | | | | | | | |
| Training dataset (n=279) | 0.463 | 0.784 | (0.729-0.840) | 0.667 | 0.815 | 0.482 | 39.8% |
| TKI_Sorafenib (n=229) | 0.346 | 0.792 | (0.732-0.852) | 0.710 | 0.713 | 0.423 | 40.6% |
| TKI_not sorafenib (n=50) | 0.408 | 0.776 | (0.641-0.911) | 0.833 | 0.687 | 0.520 | 36.0% |
| HCC cancer (n=224) | 0.314 | 0.792 | (0.731-0.853) | 0.733 | 0.694 | 0.427 | 40.2% |
| Not HCC cancer (n=55) | 0.420 | 0.775 | (0.647-0.902) | 0.762 | 0.735 | 0.497 | 38.2% |
| Testing dataset (n=121) | 0.461 | 0.890 | (0.832-0.940) | 0.836 | 0.848 | 0.684 | 45.5% |
| TKI_Sorafenib (n=105) | 0.461 | 0.896 | (0.834-0.958) | 0.826 | 0.847 | 0.673 | 43.8% |
| TKI_not sorafenib (n=16) | 0.473 | 0.841 | (0.582-1.000) | 0.889 | 0.857 | 0.746 | 56.3% |
| HCC cancer (n=102) | 0.461 | 0.898 | (0.825-0.961) | 0.841 | 0.862 | 0.703 | 43.1% |
| Not HCC cancer (n=19) | 0.494 | 0.820 | (0.596-1.000) | 0.818 | 0.875 | 0.693 | 57.9% |
| **ML 12-feature model** | | | | | | | |
| Testing dataset (n=121) | 0.417 | 0.880 | (0.818-0.942) | 0.800 | 0.848 | 0.648 | 55.4% |
| **Parsimonious 7-parameter model** | | | | | | | |
| Testing dataset (n=121) | 0.340 | 0.907 | (0.855-0.960) | 0.818 | 0.848 | 0.667 | 54.5% |

ML= machine learning; TKI=tyrosine kinase inhibitor; HCC= Hepatocellular Carcinoma; TKI_not sorafenib = imatinib, nilotinib, dasatinib and sunitinib; AUROC curve=Aurea under the Receiver Operating Characteristics Curve; Sen.=sensitivity; Spe.=specificity; CI=confidence interval

**Table S13. Comparisons of the identified factors in the prediction models obtained from this study and that in RISQ-PATH model**

| **Comparisons** | **Statistical 12-parameter model to predict newly occurrence of QTc interval prolongation in electronic medical record** |  | **Smart algorithm based on RISQ-PATH model to generate smart QT signals in electronic medical record ^1^** |  |
| --- | --- | --- | --- | --- |
| **Study design** | Retrospective cohort study |  | Retrospective cross-sectional study |  |
| **Target of prediction population** | Patients who initially treated with the listed five tyrosine kinase inhibitors (TKIs), including Bcr-Abl_i_s (imatinib, nilotinib and dasatinib) and VEGFi (sunitinib and sorafenib) in outpatient units in China Medical University Hospital during 2016 to 2020 |  | All patients visited Flemish hospitals of the Nexus network (17 hospitals) in both ambulatory and hospitalized settings with last available ECG in 2015 |  |
| **Original sample size** | 1,238 |  | 145,283+54,136  (Training + validation dataset) |  |
| **Sample size in datasets** | 279+121  (Training + testing dataset) |  | 60,208+28,400  (Training + validation dataset) |  |
| **Average age** | 63.5±12 years-old |  | 63.5±18 years-old |  |
| **Model development and validation** | | | |  |
| **Model developed approach** | backward logistic regression |  | backward logistic regression with p-value >0.157 |  |
| **Number of initial factors of interest** | 55 |  | 23 |  |
| **Final number of associated factors in the model** | 12 |  | 19 |  |
| **Performance-AUROC** | 0.89 |  | 0.702 (Q**Tc≥450(♂)/ 470(♀)ms)** |  |
| **Sensitivity** | 0.91 |  | 0.403 |  |
| **Specificity** | 0.75 |  | 0.862 |  |
| **Determine the cut-off point for a high risk of QTc based on the corresponding model** | | | | |
| **-Cut-off point** | 0.463 |  | 0.035 |  |
| **-AUC of ROC** | 0.784 |  | 0.772 |  |
| **-Sensitivity** | 0.667 |  | 0.874 |  |
| **-Specificity** | 0.815 |  | 0.462 |  |
| **-Final factor and its coefficient in the final model** | | | | |
| 1 | **Prolongation of QTc in the baseline^+^** | **1.922** | Availability of a previous ECG | -0.192 |
| 2 | **Potassium < 3.5 mmol/L** | 0.859 | **QTc on a previous ECG ≥450(male)/470(female) and <500** | **2.096** |
| 3 | **Calcium < 8 mmol/L** | 0.959 | QTc on a previous ECG >500 | 2.368 |
| 4 | Total bilirubin > 2 mg/dL | 0.275 | Age>65 years | 0.682 |
| 5 | Albumin < 3.8 g/dL | 0.365 | Availability of BMI | 0.091 |
| 6 | Check ECG in emergency room | 0.592 | BMI≥30kg/m^2^ | 0.053 |
| 7 | AST ≥ 105 IU/L | 0.479 | Availability of potassium measure | 0.332 |
| 8 | ECOG score ≥ 3 | 0.821 | **Potassium < 3.5 mmol/L** | **0.553** |
| 9 | INR > 1.5 | 0.821 | Availability of calcium measure | -0.066 |
| 10 | Other CV diseases# | 1.103 | **Calcium < 8 mmol/L** | **0.509** |
| 11 | Concurrent use medications ≥ 5 items | 0.408 | Availability of eGRF measure | -0.565 |
| 12 | **Arrythmia** | **0.589** | eGFR ≤30ml/min | 0.419 |
| 13 |  |  | Availability of CRP measure | 0.480 |
| 14 |  |  | CRP>5mg/l | 0.124 |
| 15 |  |  | History of (ischemic) cardiomyopathy and/or hypertension | 0.298 |
| 16 |  |  | **History of arrythmia** | **0.802** |
| 17 |  |  | Liver failure | 1.121 |
| 18 |  |  | Neurological disorders | 0.141 |
| 19 |  |  | Number of drugs in lilst 1 of CredibleMeds | 0.088 |
| 20 |  |  | Number of drugs in list 3 of CredibleMeds | 0.064 |
| **-Constant of the final model** | -2.039 |  | -3.970 |  |

QTc=Corrected QT Interval; AST= aspartate aminotransferase; ECOG= Eastern Cooperative Oncology Group Performance Status Scale; INR= International Normalized Ratio; ECG= Electrocardiography; BMI=body mass index; TKI=tyrosine kinase inhibitor; CRP=C-reactive protein; eGRF= estimated Glomerular filtration rate;

#Other CV diseases= those CV diseases, excluding the stand-alone CV diseases which were strongly related to QTc prolongation (i.e., arrythmia [ICD 10 as I44 - I49], ischemia heart disease, hypertension, heart failure, cardiomyopathy), were grouped together to compare.

^+^: QTc interval prolongation was defined as ≥ 450 millisecond (ms) for male patients and ≥ 470 ms for female patients.

Reference:

1. Vandael E, Vandenberk B, Vandenberghe J, et al. A smart algorithm for the prevention and risk management of QTc prolongation based on the optimized RISQ-PATH model. *Br J Clin Pharmacol.* 2018;**84(12)**:2824-2835. doi:10.1111/bcp.13740
